# 1st Joint European Conference on Therapeutic Targets and Medicinal Chemistry (TTMC 2015)

**DOI:** 10.3390/ph9010001

**Published:** 2015-12-26

**Authors:** Marc Le Borgne, Samer Haidar, Olivier Duval, Bernhard Wünsch, Joachim Jose

**Affiliations:** 1 Université de Lyon, Université Lyon 1, Faculté de Pharmacie-ISPB, EA 4446 Biomolécules Cancer et Chimiorésistances, SFR Santé Lyon-Est CNRS UMS3453-INSERM US7, 8 avenue Rockefeller, F-69373, Lyon Cedex 8, France; marc.le-borgne@univ-lyon1.fr; 2 Institute of Pharmaceutical and Medicinal Chemistry, PharmaCampus, Westfälische Wilhelms-University Münster, Corrensstr. 48, Münster 48149, Germany; shaid_01@uni-muenster.de (S.H.); wuensch@uni-muenster.de (B.W.); 3 Laboratoire des Substances d’Origine Naturelle et Analogues Structuraux, UPRES EA921, UFR des Sciences pharmaceutiques et ingénierie de la santé, Université d’Angers, 16 Bd Daviers, Angers Cedex 49045, France; olivier.duval@univ-angers.fr

**Keywords:** drug discovery, medicinal chemistry, therapeutic targets, protein protein interactions, tools

## Abstract

The European Conference on Therapeutic Targets and Medicinal Chemistry is a new two-day meeting on drug discovery that is focused on therapeutic targets and the use of tools to explore all fields of drug discovery and drug design such as molecular modelling, bioorganic chemistry, NMR studies, fragment screening, *in vitro* assays, *in vivo* assays, structure activity relationships, autodisplay. Abstracts of keynote lectures, plenary lectures, junior lectures, flash presentations, and posters presented during the meeting are collected in this report.

## 1. Aim and Scope of the Meeting

The Third ChemBioInteract Conference was organized together with the 23rd Conference of the Grouping of the Pharmacochemists of the Atlantic Arc (GP2A) under the joint header 1st European Conference on Therapeutic Targets and Medicinal Chemistry (TTMC 2015, August 26–28). TTMC 2015 was held in Münster, at the PharmaCampus of the WWU Münster (Westphalia, Germany).

The major topics of the conference TTMC were anticancer drugs, new targets, protein protein interaction inhibitors, anticancer drugs and novel tools. The scientific program included keynote (KL), plenary (PL) and junior (JL) lectures given by distinguished experts in the field, as well as oral and poster presentations by young scientists, and finally a workshop on the options of research funding on a European level. Besides being a forum for intense discussions and scientific exchange, the major aim of the conference was to provide a platform for young scientists to present their research.

## 2. Conferences

### 2.1. Selective Modulators of Multidrug ABC Transporters to Target Resistant Cancer Cells (KL01)

Attilio Di Pietro

BMSSI UMR5086 CNRS-University of Lyon, Institute of Protein Biology and Chemistry, Passage du Vercors 7, Lyon 69367, France; E-Mail: a.dipietro@ibcp.fr

Multidrug ABC (“ATP-binding cassette”) transporters are involved, upon overexpression, in chemoresistant tumors by pumping anticancer drugs out of the cells. To target the “breast cancer resistance protein” ABCG2, we have screened different series of flavonoids and derivatives, such as flavones, rotenoids and acridones, and more recently chalcones (Valdameri, G., *et al.* *J. Med. Chem.* 2012, *55*, 3193–3200; Winter, E., *et al.* *J. Med. Chem*. 2014, *57*, 2930–2941), chromones (Valdameri, G., *et al.* *J. Med. Chem.* 2012, *55*, 966–970; Winter, E., *et al.* *J. Med. Chem.* 2013, *56*, 9849–9860), and indenoindoles (Gozzi, G.J., *et al.* *J. Med. Chem*. 2015, *58*, 265–277), as inhibitors of mitoxantrone efflux from transfected HEK293 human cells and chemosensitizers of cell proliferation, to establish 3D-Quantitative Structure-Activity Relationships. Two types of selective, non-competitive, inhibitors have been characterized, either inhibiting or stimulating the basal ATPase activity. The most potent one is indeed efficient *in vivo* on *SCID* mice, xenografted with human ABCG2-transfected cells, by chemosensitizing tumor growth to the drug- substrate irinotecan (Honorat, M., *et al.* *Oncotarget* 2014, *5*, 11957–11970). These selective inhibitors constitute good drug candidates, with low intrinsic toxicity, as sensitizers of cell proliferation to conventional chemotherapeutics.

The “Multidrug Resistance Protein 1” ABCC1 is able to catalyze the efflux of either glutathione conjugates or free glutathione together with hydrophobic substrate drugs. We have identified modulators such as verapamil (Trompier, D., *et al.* *Cancer Res.* 2014, *64*, 4950–4956; Perrotton, T., *et al.* *J. Biol. Chem.* 2007, *282*, 31542–31548) mimicking substrates and inducing a fast and massive efflux of intracellular glutathione from ABCC1- overexpressing cells, leading to a selective cell death through apoptosis, due to “*collateral sensitivity*”, or hypersensitivity. The overexpressed transporter then constitutes the *Achilles’ heel* of such resistant cancer cells. Since verapamil is known for its cardiotoxic effects, we investigated other types of modulators such as xanthones (Lorendeau, D., *et al.* *ChemMedChem.* 2011, *6*, 1478–1484), flavones (Lorendeau, *et al.* *Biochem. Pharmacol.* 2014, *90*, 235) and flavonoid dimers. Glutathione efflux appeared to be necessary, but not sufficient alone, to trigger apoptosis, indicating the contribution of other partner(s) or signaling pathway(s). Such apoptosis inducers may constitute a new type of anticancer drugs operating through an original strategy aimed at selectively targeting and eliminating multidrug-resistant tumors overexpressing the ABCC1 transporter (Szakacs, G., *et al.* *Chem. Rev*. 2014, *114*, 5753–5774).

### 2.2. Targeting Acute Myeloid Leukemia with Inhibitors of the Transcription Factor c-Myb (PL01)

Karl-Heinz Klempnauer

Institute for Biochemistry, University of Münster, Münster 48149, Germany; E-Mail: klempna@uni-muenster.de

The transcription factor Myb plays a key role in the hematopoietic system and has been implicated in the development of leukemia and other human cancers. Inhibition of Myb is therefore emerging as a potential therapeutic strategy for these diseases. However, due to lack of suitable inhibitors the feasibility of therapeutic approaches based on Myb inhibition has not been explored. We have generated screening systems that permit the identification of Myb inhibitors. Initial analyses show that some of the inhibitory compounds, which we have identified, disrupt the interaction of Myb and the coactivator p300. Our work demonstrates for the first time that it is possible to inhibit Myb activity by small molecules that interfere with a crucial protein-protein interaction of Myb. Analysis of the biological effects elicited by these compounds on leukemia cell lines and primary leukemic cells suggests that they may have potential for the treatment of leukemia and, possibly, other tumors driven by deregulated Myb.

### 2.3. Search for Anti-Cancer Drugs in the Era of Personalized Medicine—The Case of Acute Myeloid Leukemia (AML) (PL02)

Stein Ove Døskeland * and Lars Herfindal

TSG-group, Department Biomedicine, Medical Faculty, University of Bergen, BBB, Jonas Lies vei 91, Bergen N-5009, Norway

* Author to whom correspondence should be addressed; E-Mail: stein.doskeland@uib.no.

AML is well suited for personalized therapy: Viable AML cells can be isolated from patients, and their DNA sequence, and mRNA, micro-RNA, and protein expression (including post-translational modifications) determined. Such data already help select the optimal existing drug for individual patients. We expect, in addition, to define novel drug targets through ongoing comparative proteome analysis (with Ø. Bruserud, HUH, Bergen, Norway and the PROBE proteomics platform) of AML cells isolated from single patients before and after they become therapy resistant.

We have developed an assay of AML cell growth and viability in 3D culture under conditions mimicking the leukemic bone marrow (Gausdal, G., *et al.* *Blood* 2008, *111*, 2866–2877; Herfindal, L., *et al.* *J. Med. Chem.* 2009, *52*, 5758–5762; Oftedal, L., *et al.* *Mar. Drugs* 2010, *8*, 2659–2762). This assay reveals AML cell sensitivity to drugs that are efficient in patients, but not in commonly used, conventional cell viability assays (Herfindal, L., *et al.* *Mol. Pharm.* 2011, *8*, 360–367). We will present new results from *in vitro* testing using this assay of: (1) novel compounds from marine toxins, (2) synthetic analogs of such compounds (with P. Rongved, University of Oslo, Norway), and (3) drug candidates produced through collaborative efforts with organic chemists (M. Le Borgne, Univ. of Lyon, France), mainly directed against protein kinases like Casein-kinase II.

We will also show data on nanocarrier-encapsulated anti-AML drugs. Nanoencapsulation was used (1) to improve the specificity of drug delivery to the cancer cells, (2) to deliver a fixed proportion of two drugs, and (3) to achieve cell entry of poorly soluble or readily metabolized drugs. Finally, manipulation the cAMP system to improve drug penetration into solid tumors and the leukemic bone marrow will be described.

### 2.4. New Anti-Cancer Drugs: What MedChem Can Offer? (PL03)

Antti Poso ^1,2^

^1^ University of Eastern Finland, School of Pharmacy, P.O. Box 1627, Kuopio 70211, Finland; E-Mail: antti.poso@uef.fi

^2^ University Hospital Tübingen Division of Translational Gastrointestinal Oncology, Otfried-Müller-Strasse10, Tübingen 72076, Germany

Clinical trials in oncology have the highest failure rate compared with any other major therapeutic areas; only 5% of compounds showing anticancer activity in preclinical development will reach markets (Hutchinson, L., *et al*. *Nat. Rev. Clin. Oncol.* 2011, *8*, 313). The severe limitations of preclinical tools, such as inadequate cancer-cell-lines in use and xenographic mouse models, are potential reasons for poor performance. At the same time most of the preclinical cancer studies (both in academia and in industry) are based on published targets, but these targets are far too often not truly related to cancer. As an example Begley and Ellis (Begley, C.G., *et al*. *Nature* 2012, *483*, 531–533) have reported that only 11% of “landmark” paper on cancer can be validated by independent studies. As a very simplified conclusion we can claim that target identification is the problem in anti-cancer research.

Anti-cancer targets are typically hypothesis derived as non-biased *in vivo* screening in relevant models hasn’t been possible. Recently a new, mosaic mouse liver cancer model has been developed, making it possible to carry out non-biased functional genetic screens to identify new anti-cancer targets (Zender, L., *et al.* *Cell* 2008, *135*, 852–864) This method allows us to screen hundreds of targets in a reasonable time frame but also we are able to validate the selected targets using mouse co-expressing oncogenic NrasG12V. While this approach is surely the best preclinical cancer screening system at the moment, there are still two major bottlenecks in this approach. The selection of screening shRNA libraries should be rationalized to yield targets, which are drugable using currently available drug discovery methods. Also shRNA methodology itself is not applicable clinically and thus we must design small drug-size molecules against these targets for any meaningful clinical usage. In this presentation I describe an approach and first results of anti-cancer drug design project where functional genetic screens are combined with medicinal and computational approaches for fast active compounds identification and optimization.

### 2.5. Heterocyclic Derivatives of 3,4,5-Trimethoxystyrene as Microtubule Binding Agents (JL01)

Thierry Lomberget

EA 4446 B2C ISPB, Université Claude Bernard Lyon 1, 8 avenue Rockefeller, Lyon 69373, France; E-Mail: thierry.lomberget@univ-lyon1.fr

Microtubule Binding Agents (MBAs) are an important category of anticancer drugs, aiming to inhibit the assembly of tubulin to microtubules or the disassembly of microtubules to tubulin, thus blocking the cell division (Dumontet, C., *et al*. *Nat. Rev. Drug Discov.* 2010, *9*, 790–803). Among all the natural products that possess such mechanism of action, combretastatin A-4 has driven the interest of many research groups, due its structural simplicity and anti-proliferative activities (Pettit, G., *et al*. *J. Med. Chem.* 2005, *48*, 4087–4099). Our contribution to the development of new MBAs was focused on the design of heterocyclic derivatives of combretastatin A-4, after replacement of the B ring (Nguyen, T.T.B., *et al*. *Bioorg. Med. Chem. Lett.* 2012, *22*, 7227–7231).

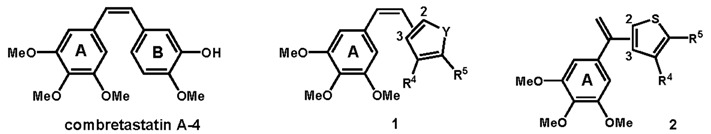


During this talk will be presented the synthesis and biological evaluation (*in cellulo* tubulin polymerization inhibition (Vassal, E., *et al*. *J. Biomol. Screen.* 2006, *11*, 377–399), anti-proliferative effects and cell cycle analysis) of 3,4,5-trimethoxystyrene derivatives **1** having various (benzo)heterocycles (Y = O, S, NR), linked at different positions. The modification of the alkene bridge was also done with the preparation of *iso* derivatives **2** having thiophene and benzothiophene rings. The most active compounds were obtained for (*Z*) stereoisomers, in the benzothiophene and methoxythiophene series.

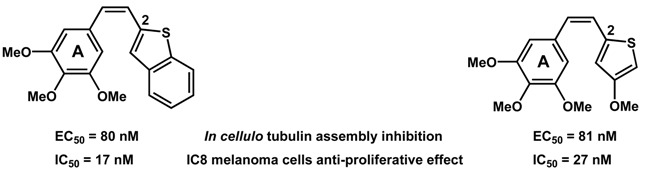


### 2.6. Targeted Drug Discovery Applied to Unmet Medical Need in Breast Cancer (PL04)

Andrew D. Westwell

School of Pharmacy and Pharmaceutical Sciences, Cardiff University, Redwood Building, King Edward VII Avenue, Cardiff, CF10 3NB, UK; E-Mail: WestwellA@cf.ac.uk

Advances in breast cancer research have established the existence of clinical disease sub-types, each with distinct pathologies, courses of disease progression, and responses to therapeutic intervention. Survival outcomes have improved dramatically in recent years for oestrogen receptor (ER)-positive disease, largely due to the routine use of tamoxifen or related anti-oestrogens such as anastrozole. On the other hand, significant disease sub-types such as “triple-negative” breast cancer (lacking expression of ER, progesterone or HER2 receptor; around 15% of cases), present a difficult challenge with standard chemotherapy having little impact on overall survival. In addition, treatment of HER2-positive breast cancer, a sub-type characterised by frequent metastatic progression, is also clinically challenging. Treatment of HER2-positive disease can be partially addressed by agents such as the monoclonal antibody trastuzumab, albeit with disease relapse and progression in many cases.

Previous research at Cardiff has established the important role of Bcl3 in metastatic progression of HER2- positive breast cancer within *in vitro* and *in vivo* models (Wakefield, A., *et al.* *Cancer Res.* 2013, *73*, 745–755). Bcl3 is a facilitator protein of the NF-kB signalling system with significant potential as a target for cancer drug design.

At the outset of this project, there were no reported inhibitors of Bcl3, however previous structural biology studies had established crystal structures for Bcl3 and protein binding partners. We chose to focus on the Bcl3- p50 protein-protein interaction, generating a pharmacophore model for virtual screening. Subsequent docking and refinement of virtual hit compounds led to the identification of ten distinct compounds for *in vitro* evaluation. One of these compounds (JS6) exhibited potent (sub-micromolar) inhibitory activity in a range of relevant breast cancer models including an NF-kB reporter cell line and in cell migration assays. Crucially, JS6 effectively suppressed metastatic progression in *in vivo* models bearing human metastatic MDA-MB-231 cells. These results have led to patent filing and subsequent licensing to Tiziana Life Sciences. Further pre-clinical development studies are ongoing and will be discussed.

### 2.7. Development of Novel Antibiotics: Synthesis and Biological Evaluation of LpxC Inhibitors (JL02)

Ralph Holl

Westfälische Wilhelms-Universität Münster, Institut für Pharmazeutische und Medizinische Chemie, Corrensstr. 48, Münster 48149, Germany; E-Mail: hollr@uni-muenster.de

Due to the growing number of multidrug resistant bacteria, the successful treatment of infectious diseases with the currently available antibiotics is becoming increasingly problematic. Therefore, novel antibiotics addressing so far unexploited bacterial targets, thereby circumventing established mechanisms of resistance, are urgently needed (Projan, S.J., *et al.* *Curr. Opin. Microbiol.* 2012, *5*, 463–465; Cooper, M.A., *et al.* *Nature* 2011, *472*, 32).

A promising strategy to combat infections caused by multidrug resistant Gram -negative bacteria is the inhibition of the Zn^2+^-dependent deacetylase LpxC, which could be validated as an antibacterial drug target. LpxC catalyzes the deacetylation of UDP-3-*O*-[(*R*)-3-hydroxymyristoyl]-*N*-acetylglucosamine, the first irreversible step of lipid A biosynthesis in Gram-negative bacteria. Lipid A, representing the hydrophobic membrane anchor of lipopolysaccharide in the outer membrane of Gram-negative bacteria, is essential for the growth and viability of the majority of Gram-negative bacteria. As the inhibition of the biosynthesis of lipid A is lethal to these bacteria, LpxC inhibitors represent a promising new class of antibiotics (Raetz, C.R.H., *et al.* *Annu. Rev. Biochem.* 2007, *76*, 295–329; Barb, A.W., *et al.* *Curr. Pharm. Biotechnol.* 2008, *9*, 9–15).

The potent LpxC inhibitor CHIR-090, which contains a hydroxamate moiety to chelate the catalytic Zn^2+^-ion and a hydrophobic region mimicking the fatty acyl chain of the natural substrate, was chosen as lead compound for the development of *C*-furanosidic and proline-derived LpxC inhibitors as well as of the respective open-chain derivatives (Barb, A.W., *et al.* *Biochemistry* 2007, *46*, 3793–3802). To access the envisaged compounds, chiral-pool syntheses were elaborated. The inhibitory activity of the synthesized compounds was determined in an LpxC enzyme assay and disc diffusion assays against various clinically important Gram-negative bacteria were performed to reveal their antibacterial properties. For the establishment of structure-activity relationships, the length and structure of the lipophilic side chain of the synthesized LpxC inhibitors as well as the stereochemistry of the compounds were especially taken into account (Löppenberg, M., *et al.* *Org. Biomol. Chem.* 2013, *11*, 6056–6070; Szermerski, M., *et al.* *Bioorg. Med. Chem.* 2014, *22*, 1016).

### 2.8. NMR for Fragment-Based Drug Design (JL03)

Isabelle Krimm

Institute of Analytical Sciences, CNRS, University of Lyon, 5 rue de la Doua, Villeurbanne 69100, France; E-Mail: isabelle.krimm@univ-lyon1.fr

Nuclear Magnetic Resonance is a powerful technique for probing and characterizing protein-ligand interactions. NMR is particularly robust for fragment screening, generating a low rate of false positives and false negatives. Well-known techniques include the ligand-observed NMR experiments Saturation transfer Difference and Waterlogsy. Transferred Interligands NOES (referred as INPHARMA), can also be used to optimize a protein inhibitor. In addition, structural information can be inferred from 1D 1H NMR experiments such as STD. The latter are shown to be very useful for (i) assessing the binding mode of the ligand; (ii) ranking a series of related ligands; and (iii) discriminating particular ligand orientation upon protein binding from non-specific protein binding. Protein-observed NMR experiments, in particular the well-known 2D HSQC/HMQC experiments, are typically used for ligand screening and measurement of protein-ligand affinities. We show here that these experiments can be used for assessing the binding modes of the ligands. In this approach, the experimental chemical shift perturbations of protein resonances observed upon ligand binding are compared to theoretical chemical shift perturbations calculated for protein-ligand structures obtained by docking. The docking structures that display the best agreement between experimental and back-calculated data are shown to be the experimental protein-ligand structure.

### 2.9. Multiple Sites to Target Protein Kinase CK2 (PL05)

Karsten Niefind

Department of Chemistry, Institute of Biochemistry Cologne, University of Cologne, 50674 Cologne, Germany; E-Mail: karsten.niefind@uni-koeln.de

Protein kinase CK2 is an essential serine/threonine kinase with a broad and acidophilic substrate profile and particular heterotetrameric architecture: the CK2 holoenzyme is composed of two separate catalytic subunits (CK2α) attached to a dimer of non-catalytic chains (CK2β).

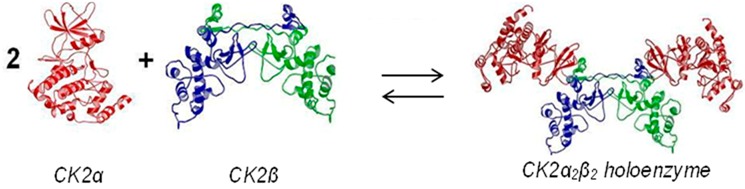


CK2 activity is ubiquitously present in eukaryotic cells but significantly overexpressed in rapidly proliferating tissues, especially in tumour cells. Therefore, the enzyme is subject of pharmaceutical drug design efforts. The catalytic subunit CK2α offers several binding sites which are naturally addressed by (co-)substrate molecules, CK2β or other important factors. These sites (and the corresponding interactions) can be compromised artificially alone or in combination. In particular interfering with the unique CK2α/CK2β interaction seems to be an attractive strategy to develop highly selective inhibitors. These concepts will be corroborated in the talk by structural, biophysical and enzymological data.

### 2.10. The Secretory Machinery in Endothelial Cells Regulating Haemostasis and Local Inflammation (PL06)

Volker Gerke

Institute of Medical Biochemistry, Centre for Molecular Biology of Inflammation, University of Muenster, Münster 48149, Germany; E-Mail: gerke@uni-muenster.de

Endothelial cells regulate thrombosis, haemostasis and local inflammatory responses by supplying the vasculature with a number of factors that include the pro-coagulant and pro-inflammatory von-Willebrand factor (vWF) and P-selectin. Both proteins are stored in large organelles, the Weibel-Palade bodies (WPB), and can be secreted in a Ca^2+^-regulated manner following endothelial activation. The molecular mechanisms underlying WPB biogenesis and acute WPB exocytosis are far from being understood although a number of endothelial proteins involved in this process have been described. These include members of the SNARE and annexin families that most likely participate in docking of WPB at the plasma membrane and initiating the actual fusion event. Recent progress towards the identification of the endothelial machinery that supports WPB maturation and exocytosis will be discussed.

### 2.11. Some Like it Hot—Biomolecule Analytics Using Microscale Thermophoresis (MST) (JL04)

Christian Kleusch

NanoTemper Technologies GmbH, Munich 81369, Germany; E-Mail: Christian.kleusch@nanotemper.de

The technology is called “Microscale Thermophoresis”, which means that we measure the directed motion of molecules along a local temperature gradient generated with infrared laser radiation. Thermophoresis depends on size, charge and solvation entropy of the molecules in solution. Since one of the parameters changes in virtually every binding event, we can measure protein-protein, protein-nucleic acid, and protein-ribosome interactions. Technology allows even measurement of the interactions of small molecules (drugs, sugars, ions) with proteins. Measurements require less than 5 µL of sample volume at nanomolar concentrations and takes just 10 min. The method is also suited for the measurement in complex biological liquids as serum or cell lysate and can detect aggregates in the sample. Orthogonal methods include Surface Plasmon Resonance and Isothermal Titration Calorimetry.

The presentation will cover: (1) Technical details and benefits of the Microscale Thermophoresis technology platform and (2) Examples of interaction measurements ranging from protein–ribosome, protein–protein, small molecule–receptor down to protein–ion binding studies to experiments where the interactions between receptors incorporated in vesicles and soluble proteins are analyzed.

### 2.12. Predicting Orphan Allosteric Binding Sites (PL07)

Holger Gohlke

Institute of Pharmaceutical and Medicinal Chemistry, Heinrich-Heine-University, Düsseldorf 40225, Germany; E-Mail: gohlke@uni-duesseldorf.de

A quantitative description of allostery is fundamental to an understanding of processes in living systems and of practical relevance when developing allosteric modulators. More recent models of allostery stress the influence of dynamics and large-scale conformational disorder. This provides the challenge from a computational point of view to develop an efficient methodological framework for analyzing, understanding, and predicting allostery in dynamic systems.

Here we present Constraint Network Analysis (Pfleger, C., *et al.* *J. Chem. Inf. Model.* 2013, *53*, 1007–1015; Rathi, P.C., *et al.* *Bioinformatics* 2015, *31*, 2394–2396) (CNA, web server (Krüger, D.M., *et al.* *Nucleic Acids Res.* 2013, *41*, W340): http://cpclab.uni-duesseldorf.de/cna/) as such a framework. CNA applies concepts grounded in rigidity theory to analyze biomolecular flexibility (Pfleger, C., *et al.* *J. Comput. Chem.* 2012, *34*, 220–233). The approach works on conformational ensembles (Rathi, P.C., *et al.* *J. Biotechnol.* 2012, *159*, 135–144) or ensembles of network topologies (Pfleger, C., *et al.* *Structure* 2013, *21*, 1725–1734), that way considering thermal motions and, hence, dynamics of molecules in an efficient way.

We applied CNA in terms of a perturbation approach to gain structure-based insights into allosteric signaling and coupling in dynamic proteins. Validating the approach against NMR relaxation data for the system Eglin c shows that is correctly identifies contiguous pathways of allosteric coupling and accurately predicts the magnitude of coupling energies. When applied to the therapeutic target PTP1B, predicted pathways of allosteric coupling cover residues of functional importance, and when applied to the adhesion protein LFA-1 involved in immunobiology, the approach correctly predicts the sign of the cooperative coupling.

In all, this demonstrates that the approach can describe quantitatively allostery in dynamic systems. Finally, we extended our approach to allow for the identification of sites in proteins that are allosterically coupled even in cases when no allosteric modulator is known yet. This makes CNA an interesting tool in the context of target identification and validation.

### 2.13. Synthesis and Biochemical Evaluation of Ethanoanthracenes as Cytotoxic Agents in Burkitt’s Lymphoma (PL08)

Andrew J. Byrne ^1^, Sandra A. Bright ^2^, D. Clive Williams ^2^ and Mary Jane Meegan ^1,^*

^1^ School of Pharmacy and Pharmaceutical Sciences, Trinity Biomedical Sciences Institute, Trinity College Dublin, Dublin 2, Ireland

^2^ School of Biochemistry and Immunology, Trinity Biomedical Sciences Institute, Trinity College Dublin, Dublin 2, Ireland

* Author to whom correspondence should be addressed; E-Mail: mmeegan@tcd.ie.

Cancers of the lymphatic cells (lymphomas) account for approximately 12% of malignant diseases worldwide (Blum, K.A., *et al.* *Blood* 2004, *104*, 3009–3020). Burkitt’s lymphoma (BL) is a non-Hodgkin’s lymphoma (NHL), which manifests as tumours composed of small non cleaved B-cell lymphocytes (Cloonan, S.M., *et al.* *Int. J. Cancer* 2011, *128*, 1712–1723). We have sought to identify possible alternatives to the current clinical drugs used in the treatments for lymphomas and leukaemias. Our previous research has demonstrated promising antiproliferative activity of antidepressants maprotiline (a NET selective antidepressant) and fluoxetine. We have also screened amphetamine related, synthesis derived compounds in Burkitt’s lymphoma (Cloonan, S.M., *et al.* *Leuk. Lymphoma* 2010, *51*, 523–539; McNamara, Y.M., *et al.* *Bioorg. Med. Chem.* 2011, *19*, 1328–1348). The most important finding was caspase independent type-2 autophagic cell death that was induced in the chemoresistant BL DG-75 cells, which exhibited low EC50 values in the ranges of 5.9–15.3 µM and 5.9–15.6 µM when treated with maprotiline and fluoxetine respectively over 72 h period (Cloonan, S.M., *et al.* *Leuk. Lymphoma* 2010, *51*, 523–539; McNamara, Y.M., *et al. Bioorg. Med. Chem.* 2011, *19*, 1328–1348).

The screening of a diverse library of compounds structurally related to amphetamines and maprotiline and was further investigated in BL and CLL cell lines (McNamara, Y.M., *et al.* *Eur. J. Med. Chem.* 2014, *71*, 333–353). A number of 9,10-dihydro-9,10-ethanoanthracenes were found to reduce cell viability to a greater extent than maprotiline in BL cell lines. In addition, related 9-substituted anthracenes were found to exert a potent caspase-dependant apoptotic effect in the BL cell lines, while having minimal effect on the viability of peripheral blood mononuclear cells (PBMCs). Compounds also displayed activity in multi-drug resistant (MDR) cells. Activity of compounds was demonstrated to be superior in antiproliferative and pro-apoptotic effects in CLL cell lines to the current treatment options for CLL e.g., fludarabine.A common structural motif was identified in the compounds exhibiting the most potent effects as pro-apoptotic agents.

## 3. Flash Presentations

### 3.1. Assay Development for NAD^+^-Dependent Deacylases (Sirtuins)

Sören Swyter ^1,^*, Tobias Rumpf ^1^, Oliver Einsle ^2^ and Manfred Jung ^1^

^1^ Institut für Pharmazeutische Wissenschaften, Albert-Ludwigs-Universität, Albertstraße 25, Freiburg 79104, Germany

^2^ Institut für Biochemie, Albert-Ludwigs-Universität, Albertstraße 25, Freiburg 79104, Germany

* Author to whom correspondence should be addressed; E-Mail: soeren.swyter@pharmazie.uni-freiburg.de.

Sirtuins are an evolutionary conserved family of NAD^+^-dependent Lysine deacylases (KDAC) (Trapp, J., *et al*. *Curr. Drug Targets* 2006, *7*, 1553–1560). There are seven human isotypes which differ in their subcellular localization, their enzymatic activities as well as in their deacylation substrates. The main enzymatic activity of Sirt1-3 is the deacetylation. They deacetylate a wide range of protein substrates such as p53, α-tubulin or acetyl-CoA-synthetase. A dysregulation of the cellular acetylation level has been associated with human diseases e.g., cancer, neurodegerenative and metabolic diseases which makes a modulation of sirtuin activity a promising strategy for pharmaceutical intervention (Hoffmann, G., *et al*. *J. Biol. Chem.* 2014, *289*, 5208–5216; Feldman, J.L., *et al*. *J. Biol. Chem.* 2013, *288*, 31350–31356).

For the screening of potential modulators of sirtuins, a well functional assay system feasible for high throuput-screening (HTS) is indispensable. So far, there are some well established assay systems for measuring the binding or activity potential of sirtuins in complex with ligands. However, all these protocols dealing with labeled substrate or are challenging in the performance for HTS (Heltweg, B., *et al*. *Methods* 2005, *36*, 332–337; Schiedel, M., *et al*. *J. Biomol. Screen.* 2015, *20*, 112–121). Here we present the development of a homogeneous, label free assay bases on a peptide substrate for Sirt2 & 3.

### 3.2. Benzhydryl-1H-1,2,4-Triazole and 1-Benzhydryl-1H-Imidazole Analogues of Phenstatin as Dual Tubulin and Aromatase Targeting Anticancer Agents

Gloria Ana and Mary Jane Meegan *

School of Pharmacy and Pharmaceutical Sciences, Trinity Biomedical Sciences Institute, Trinity College Dublin, Dublin 2, Ireland

* Author to whom correspondence should be addressed; E-Mail: mmeegan.tcd.ie.

Phenstatin is a synthetic antitumour benzophenone, related in structure to the combretastatin, which demonstrates strong antitubulin activity by binding to the colchicine binding site of tubulin (Pettit, G., *et al*. *J. Med.* 1998, *41*, 1688–1695). Letrozole is a third generation aromatase inhibitor characterised by the presence of the triazole heterocycle (Altundag, K., *et al*. *Oncologist* 2006, *11*, 553–562). This project describes the synthesis, characterization and biochemical evaluation of a library of hybrid compounds containing both the trimethoxyaryl pharmacophore of phenstatin and the triazole of letrozole with potential dual action as aromatase and tubulin inhibitors.

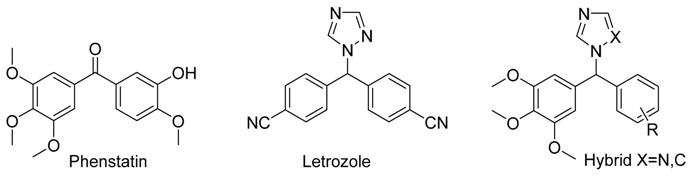


The chemistry involved in the synthesis of these compounds requires a reaction between a 3,4,5-trimethoxyaryl bromide and substituted aryl aldehydes under strongly basic conditions which leads to the formation of the secondary alcohol as a common intermediate of the two series of compounds. For the addition of the triazole the secondary alcohol was reacted in an open vessel microwave with the use of a Dean-Stark trap to eliminate the water formed during the reaction (Veillet. S., *et al*. *Anal. Chim. Acta* 2009, *632*, 203–207). A library of related compounds containing the imidazole heterocycle has also been synthesised. For the introduction of imidazole, the secondary alcohol was reacted with carbonyldiimidazole in dry acetonitrile at reflux (Njar, V. *Synthesis* 2000, 2019–2028). All the novel synthesised compounds have been characterised (^1^H-NMR, ^13^C-NMR, IR, HRMS). The purity of the final products have also been evaluated by HPLC. The structures of the novel compounds were established by single crystal X-Ray analysis. The newly synthesised benzyhydryl-1*H*-1,2,4-triazole and 1-benzhydryl-1*H*-imidazole analogues of phenstatin are now characterised and preliminary biochemical results (antiproliferative assays with MCF-7; human breast cancer cell line and HL-60; human leukemia cell line) will be presented together with rationalization of the structure-activity relationships for the series.

### 3.3. An Autodisplay Based Screening Assay for the Identification of Inhibitors Targeting the Dimerization Domain of Human Chaperone HSP90

Bertan Bopp ^1,^*, Emanuele Ciglia ^2^, Anissa Ouald-Chaib ^2^, Georg Groth ^3^, Holger Gohlke ^2^ and Joachim Jose ^1^

^1^ Institute of Pharmaceutical and Medicinal Chemistry, Westfälische Wilhelms-Universität, Pharma Campus, Corrensstraße 48, Münster 48149, Germany

^2^ Institute of Pharmaceutical and Medicinal Chemistry, Heinrich-Heine-University Düsseldorf, Universitätsstr. 1, Düsseldorf 40225, Germany

^3^ Institute for Biochemical Plant Physiology, Heinrich-Heine-University Düsseldorf, Universitätsstr. 1, Düsseldorf 40225, Germany

* Author to whom correspondence should be addressed; E-Mail: Bertan.Bopp@uni-muenster.de.

Human Hsp90 (Hsp90) is a homodimeric chaperone, essential for the maturation of numerous proteins. Some of these proteins are involved in tumor formation and growth, which makes Hsp90 an interesting drug target for cancer treatment (Wegele, H., *et al*. *Rev. Physiol. Biochem. Pharmacol.* 2004, *151*, 1).

Here, we describe a novel autodisplay based method to screen for small molecules that inhibit PPI. The autodisplay technology was used to express the chaperone Hsp90 on the surface of *Escherichia coli*. Functional folding and dimerization was confirmed by binding of FITC-labeled p53, a natural client protein of Hsp90 and subsequent analysis by flow cytometry. By computational analysis hot spots in the C-terminal domain of Hsp90 important for dimerization were identified (Ciglia, E., *et al*. *PLoS ONE* 2014, *9*, e96031). These hot spot predictions were used to design peptides that were supposed to inhibit Hsp90 dimerization. Because binding of FITC-labeled p53 is dependent on Hsp90 dimer formation, a loss of fluorescence in the flow cytometer analysis indicates inhibition. This reduction in fluorescence turned out to be dose-dependent with respect to the inhibitor concentration, and an IC50 of 8.96 µM could be determined for the best inhibitor H3. By microscale thermophoresis measurement with the purified C-terminal domain of Hsp90 it was verified that H3 indeed binds the C-terminal domain of Hsp90 with a KD value of 1.46 µM. Up to now, H3 is the first inhibitor shown to target the C terminal dimerization domain of Hsp90 with a KD value in the low micromolar range (Bopp, B., *et al*. *Biochem Biophys Acta*, 2015, unpublished results).

### 3.4. Synthesis and Biological Evaluation of 1-Methyl-1,6-naphthyridin-2(1H)-One Derivatives as Potent HSP90 C-Terminal Inhibitors

David Montoir ^1^, Eléonore Lepvrier ^2^, Alain Tonnerre ^1^, Cyrille Garnier ^2^, Sophie Barillé-Nion ^3^, Muriel Duflos ^1^ and Marc-Antoine Bazin ^1,^*

^1^ Université de Nantes, Nantes Atlantique Universités, Laboratoire de Chimie Thérapeutique, Cibles et Médicaments des Infections et du Cancer, IICiMed EA 1155, UFR des Sciences Pharmaceutiques et Biologiques, 1 rue Gaston Veil 44035 Nantes Cedex, France

^2^ Université de Rennes 1, Institut de Génétique et Développement de Rennes, UMR-CNRS 6290, Campus Beaulieu Bâtiment 13, 263 avenue du Général Leclerc 35042 Rennes Cedex, France

^3^ CRCNA, UMR 892 INSERM/6299 CNRS/Université de Nantes, Team 8 ‘‘Cellsurvival and tumor escape in breast cancer’’, Institut de Recherche en Santé de l’Université de Nantes, IRS-UN, 8 quai moncousu, 44000 Nantes Cedex, France

* Author to whom correspondence should be addressed; E-Mail: marc-antoine.bazin@univ-nantes.fr.

The 90-kDa Heat shock protein (Hsp90) is an ATP-dependent chaperone known to play a crucial role in protein homeostasis. Hsp90 is directly involved in the conformational stability of numerous oncogenic proteins (e.g., Her2, Raf1, Akt, *etc.*), many of which are associated with cancer cell survival (Garcia-Carbonero, R., *et al.* *Lancet Oncol*. 2013, *14*, 358–369; Zhao, H., *et al.* *Eur. J. Med. Chem.* 2015, *89*, 442–466). Novobiocin, an aminocoumarin antibiotic, was reported as the first Hsp90 C-terminal inhibitor. However, due to its low antiproliferative activity (IC_50_ = 700 μM in SKBr3 breast cancer cell line), it was considered unsuitable for therapeutic application. Subsequently, many studies have led to the development of novobiocin analogues which manifest low micromolar activity (Kusuma, B.R., *et al.* *Bioorg. Med. Chem*. 2014, *22*, 1441–1449).

Prior studies have shown that attachment of a benzamide side chain to the 3-position of the coumarin ring resulted in a significant enhancement in antiproliferative activity while noviose at the 7-position could be replaced by hydrophilic moiety maintaining or increasing the biological activity (Zhao, H., *et al.* *ACS Med. Chem. Lett*. 2014, *5*, 84–88).

In this context, we focused our work on the synthesis of new analogues of novobiocin derived from the 1-methyl-1,6-naphthyridin-2(1*H*)-one scaffold. The target compounds were obtained in two steps from a key amine intermediate, the 3-amino-7-chloro-1-methyl-1,6-naphthyridin-2(1*H*)-one, which was converted into amide at position 3, and functionalized with amines at position 7.

Newly synthesized compounds were evaluated against two breast cancer cell lines and selected regarding their ability to bind the mammalian Hsp90.

### 3.5. Quantitative Elemental Bioimaging of a Palladium-Tagged Photosensitizer in Tumor Spheroids

Ann-Christin Niehoff ^1,2^, Aline Moosmann ^3^, Judith Söbbing ^4^, Arno Wiehe ^5^, Dennis Mulac ^4^, Sylvia Wagner ^3^, Michael Sperling ^1^, Hagen von Briesen ^3^, Klaus Langer ^4^ and Uwe Karst ^1,^*

^1^ Institute of Inorganic and Analytical Chemistry, University of Münster, Corrensstr. 30, Münster 48149, Germany

^2^ NRW Graduate School of Chemistry, University of Münster, Münster 48149, Germany

^3^ Department of Cell Biology and Applied Virology, Fraunhofer Institute of Biomedical Engineering, Ensheimer Str. 46, St. Ingbert 66386, Germany

^4^ Biolitec Research GmbH, Otto-Schott-Str. 15, Jena 07745, Germany

^5^ Department of Pharmaceutical Technology and Biopharmacy, University of Münster, Corrensstr. 48, Münster 48149, Germany

* Author to whom correspondence should be addressed; E-Mail: uk@uni-muenster.de.

Photosensitizers are frequently used as drugs in photodynamic therapy. Due to accumulation of the photosensitizer within malignant tissue and subsequent illumination with light of a specific wavelength, highly reactive oxygen species arise, which are able to induce apoptosis of the tumor cells. The distribution of the photosensitizer in tissues can be monitored by fluorescence microscopy. However, the major restrictions of this technique are its high limits of detection. Laser ablation coupled to inductively coupled plasma mass spectrometry offers much higher sensitivity enabling the investigation of the fate of the photosensitizers. Tagging of the drugs with Pd enabled the detection by means of ICP-MS.

The distribution of Pd in biological matrices was determined. Spheroidal cell cultures were used as a model system for malignant tissues and were incubated with different concentrations of the Pd-tagged photosensitizer 5,10,15,20-tetrakis(3-hydroxyphenyl)porphyrin (mTHPP). Additionally, effects of the dosage form of the photosensitizer were elucidated by incubation of the tumor spheroids with the pure substance and poly(lactic-co-glycolic acid) (PLGA) nanoparticles. The incubated cells were embedded and sliced into 5 µm thin cryosections. The cellular uptake and intracellular accumulation of the drugs was investigated by visualization of the distribution of Pd. Thus, images with a lateral resolution of 10 µm were generated. The enrichment of the drug occurs within the first cell layers of the spheroid. In case of incubation with the pure substance, an accumulation of the drug in specific areas can be shown, while the nanoparticles are distributed more homogeneously (Niehoff, A.-C., *et al.* *Metallomics* 2014, *1*, 77–81).

### 3.6. The Receptor Tyrosine Kinase Ron as Therapeutic Target in Ewing Sarcoma Metastases

Carolin Schleithoff ^1,3,^*, Birgit Lechtape ^1^, Claudia Tulotta ^2^, Amelie Tillmanns ^1^, Christiane Schaefer ^1^, Uta Dirksen ^1^, Jenny Potratz ^1^ and Georg Hempel ^3^

^1^ Pediatric Hematology/Oncology, University Children’s Hospital Münster, Albert-Schweitzer-Campus A1, Münster 48149, Germany

^2^ Institute ofBiology Leiden, University Leiden, Einsteinweg 55, Leiden 233 CC, The Netherlands

^3^ Institute of Pharmaceutical and Medicinal Chemistry, WestfälischeWilhelms-UniversitätMünster, Corrensstaße 48, 48149, Germany

* Author to whom correspondence should be addressed; E-Mail: c.schleithoff@outlook.de.

Ewing sarcoma is the second most common bone cancer in children and survival rates of metastatic disease remain poor. Receptor tyrosine kinases (RTKs) are cell-surface proteins regulating cellular migration, proliferation and survival. IGF1R is a RTK involved in Ewing Sarcoma development and its therapeutic inhibition has shown promising preclinical results. Unfortunately resistances occurred implying alternative RTK signaling. RTK recepteurd’originenantais (RON) has shown its potential to overcome such resistances. The aim of this project was to validate RTK RON as therapeutic target in Ewing Sarcoma, alone and in combination with IGF1R inhibition.

RON is expressed and activated in sarcoma cell lines. Activation patterns changed during FBS starvation and RON ligand MSP alone did not lead to further RON activation suggesting different activation mechanisms. RNAi experiments revealed a role for RON in cell migration, colony-forming potential *in vitro* and survival and extravasation in a zebrafish model *in vivo*. Therapeutic inhibition by a monoclonal antibody lead to reduced cell migration *in vitro*, but had little impact on cell proliferation. Co-inhibition of RON and IGF1R did not overcome resistances (Pierce, L.T., *et al*. *Eur. J. Med. Chem.* 2012, *56*, 292).

Several RON isoforms were detected in Ewing Sarcoma cell lines and in 11 of 19 patient samples. Notably shortform RON (sfRON) is a constitutively active RON isoform which lacks the extracellular, antibody binding domain. The small molecule tyrosine kinase inhibitor BMS-777607 binds intracellular the kinase domain and showed some activity on proliferation and migration of Ewing Sarcoma cell lines.

The receptor tyrosine kinase RON is involved in Ewing sarcoma cell migration. This fact supports the relevance of RON as a therapeutic target. RON isoforms were shown to have great impact on the targeting strategy.

**Acknowledgements**: Financial supported by the Deutsche Krebshilfe (109567).

### 3.7. Strategies to Select the Right Indeno[1,2-b]indole Derivative(s) for ABCG2-Targeting in Vivo Assays

Nathalie Guragossian ^1^, Gustavo Jabor Gozzi ^2^, Zouhair Bouaziz ^1^, Evelyn Winter ^2^, Glaucio Valdameri ^2^, Raphael Terreux ^3^, Christelle Marminon ^1^, Ahcène Boumendjel ^4^, Joachim Jose ^5^, Attilio Di Pietro ^2^ and Marc Le Borgne ^1,^*

^1^ EA 4446 B2C, ISPB, Université Claude Bernard Lyon 1, 8 avenue Rockefeller, IBCP, Lyon 69373, France

^2^ Equipe Labellisée Ligue 2014, BMSSI UMR 5086 CNRS/Université Lyon 1, IBCP, Lyon 69373, France

^3^ Bioinformatique structures et interactions, BMSSI UMR 5086 CNRS/Université Lyon 1, IBCP, Lyon 69373, France

^4^ University Grenoble Alpes/CNRS, DPM UMR 5063, Grenoble F-38041, France

^5^ Institute of Pharmaceutical and Medicinal Chemistry, PharmaCampus, Westfälische Wilhelms-University Münster, Corrensstr. 48, Münster 48149, Germany

* Author to whom correspondence should be addressed; E-Mail: marc.le-borgne@univ-lyon1.fr.

ABCG2 is a 72kDa sub-family G member 2 of ATP-binding cassette membrane proteins that functions as an efflux pump against toxins and xenobiotics, conferring cross-resistance to several classes of anticancer chemotherapeutics. Development of *ABCG2 inhibitors* can be used in combination with anticancer drugs to block the drug secretion from cancer cells.

Functionalized indeno[1,2-*b*]indoles are compounds that have already shown a good inhibitory activity against the protein Casein Kinase 2 (CK2) (Hundsdörfer, C., *et al.* *Bioorg. Med. Chem.* 2012, *20*, 2282–2289) and be converted into ABCG2 inhibitors with an IC_50_ in the submicromolar range upon specific substitutions on the C- and/or D-ring(s) (Gozzi, G.J., *et al.* *J. Med. Chem.* 2015, *58*, 265–277; Gozzi, G.J. *et al.* *Drug Des. Dev. Ther.* 2015, *9*, 3481–3495). From such a work, some selected indeno[1,2-*b*]indoles (both ketonic and phenolic derivatives) with high therapeutic index on cultured cells have been discriminated as promising candidates for *in vivo* evaluation.

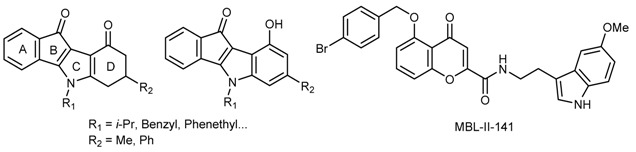


*In silico* approaches predicting ADME and toxicity properties (ACD Percepta v2012, QSAR Toolbox 3.3 softwares) were used to select the best compounds, while excluding those with low bioavailability and/or high acute or chronic toxicity. In parallel, a bimodulation assay with a highly-active ABCG2 chromone derivative inhibitor (MBL-II-141) (Honorat, M., *et al.* *Oncotarget* 2014, *5*, 11957–11970) was performed to sensitize tumor growth to the ABCG2 substrate irinotecan. We propose an integrated approach (*in silico* ADMET/bimodulation) to help the selection of best indenoindole derivatives for further biological explorations.

### 3.8. Access to New Aminoquinoline Antimalarial Drugs Using an Enantioselective Sharpless Aminohydroxylation

Guillaume Bentzinger, Alexandra Dassonville-Klimpt and Pascal Sonnet

Laboratoire LG2A, CNRS FRE 3517, UFR de pharmacie, 1 rue des Louvels, Université Picardie Jules Verne, 80037 Amiens cedex 1, France

* Author to whom correspondence should be addressed; E-Mail: guillaume.bentzinger@u-picardie.fr.

Malaria, due to a *Plasmodium* protozoan, is the 5th most lethal infection in the world (*World Malaria Report*; WHO: Geneva, Switzerland, 2009). The emergence of drug resistance continues to be a serious global problem. New antimalarial drugs are needed and this is why our team is involved in the design and synthesis of new antimalarial compounds. Extensive work has been done to synthesize chloroquine analogs but with much less in regard to mefloquine derivatives. Consequently, mefloquine **1** and its derivatives still remain very attractive synthetic targets. Recently, we have described the asymmetric synthesis and the biological activity ofaminoquinolinemethanols **2** (Jonet, A., *et al*. *Tetrahedron: Asymmetry* 2011, *22*, 138). Some structure-activity relationship have been highlighted: (i) importance of the absolute configuration of asymmetric carbons; (ii) importance of amines (aliphatic *vs*. aromatic). The most active molecule synthesized, in this series, is the aminoquinolinemethanol **3** with a *S* configuration and a pentyl group (IC_50_ = 6.98 nM) on a chloroquine-resistant W2 strain. We want now study the influence on the biological activity of the position of the amino group and the alcohol group. We present here a synthesis of aminoquinolinethanols **4** through an enantioselective Sharpless aminohydroxylation reaction.

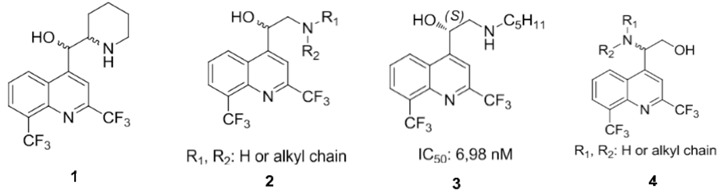


### 3.9. Selective Functionalization of the Indazole Scaffold, Application to the Synthesis of 5-HT_7_R Spect Radiotracers

Yohann Berhault *, Bao-Vy Lam, Christine Fossey, Silvia Stiebing, Thomas Cailly, Valérie Collota and Frédéric Fabis

CERMN, EA-4258, FR CRNS 3038, Université de Caen, UFR des Sciences Pharmaceutiques, Boulevard Becquerel, 14032 Caen cedex, France

* Author to whom correspondence should be addressed; E-Mail: yohann.berhault@unicaen.fr.

Serotonin (5-hydroxytryptamine, 5-HT) is a neurotransmitter acting on the central nervous system and peripheral tissues through a large variety of receptors. The human 5-HT7 receptor (5-HT7R) is the last 5-HT receptor subtype identified by Bard in 1993 (Bard, J., *et al*. *J. Biol. Chem.* 1993, *268*, 23422–23426; Charnay, Y., *et al*. *Dialogues Clin. Neurosci.* 2010, *12*, 471–487). Although several selective ligands for the 5-HT7R have been described, to date no efficient radiotracers have been discovered.

By analogy with recent literature (Leopoldo, M., *et al*. *Pharmacol. Ther.* 2011, *129*, 120–148) and preliminary works performed in our laboratory (Paillet-Loilier, M., *et al*. *Bioorg. Med. Chem. Lett.* 2007, *17*, 3018–3022), we are interested in the development of iodinated 5-HT7R ligands in the indazole series to design potential radiotracers for imaging studies of this receptor using Single Photon Emission Computed Tomography (SPECT). In order to get a convergent and straightforward access to iodinated analogues, our group has developed two methodologies:

1. The selective functionalization of position 3 of indazole to introduce a thioalkyl chain using a one pot procedure.
2. A Pd-catalysed C-H iodination of the 1-arylindazole in order to access iodinated compounds. These two methodologies have been used to provide polyfunctional indazoles within minimum steps

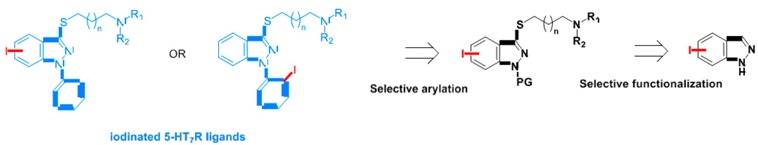

### 3.10. Inhibition of C/EBPb by the Sesquiterpene Lactone Helenalinacetate

Anke Jakobs ^1,^*, Sagar Uttarkar ^1^, Joachim Jose ^2^, Carsten Müller-Tidow ^3^, Thomas J. Schmidt ^4^, Karl-Heinz Klempnauer ^1^

^1^ Institute for Biochemistry, Westfälische Wilhelms-Universität Münster, Wilhelm-Klemm-Straße 2, Münster 48149, Germany

^2^ Institute for Pharmaceutical and Medical Chemistry, Westfälische Wilhelms-Universität Münster, Corrensstraße 48, Münster 48149, Germany

^3^ Department of Medicine A, Universitätsklinikum Halle (Saale), Ernst-Grube-Straße 40, Halle (Saale) 06120, Germany

^4^ Institute for Pharmaceutical Biology and Phytochemistry, Westfälische Wilhelms-Universität Münster, Corrensstraße 48, Münster 48149, Germany

* Author to whom correspondence should be addressed; E-Mail: a_jako02@uni-muenster.de.

The proto-oncogene c-Myb encodes a transcription factor (c-Myb) which is highly expressed in progenitor cells of the hematopoietic system, where it regulates the expression of genes involved in the lineage determination, proliferation and differentiation. c-Myb cooperates with C/EBPβ to activate transcription of different myeloid-specific genes (Burk, O., *et al*. *EMBO J.* 1993, *12*, 2027–2038). c-Myb is emerging as an interesting therapeutic target because its deregulation is involved in the development of different types of leukemia and other human tumors (Ramsay, R.J., *et al*. *Nat. Rev. Cancer* 2008, *8*, 523–534). We have developed a fluorescence-based test system that enables the screening of compounds that have the ability to interfere with the activation of c-Myb target genes (Bujnicki, T., *et al*. *Leukemia* 2012, *26*, 5–622). Using this system to screen the inhibitory activity of sesquiterpene lactones, a class of compounds that are the active components of a variety of medicinal plants (Schmidt, T.J., *et al*. *Curr. Org. Chem.* 1999, *3*, 577–605; Schomburg, C., *et al*. *Eur. J. Med. Chem.* 2013, *63*, 313–320), we have identified helenalin acetate as a new potent inhibitor of the Myb-dependent target gene expression. We have now studied the molecular mechanism underlying its inhibitory mechanism. Our results indicate that the observed inhibition is due to direct inhibition of C/EBPβ. Our compound is the first highly potent inhibitor of C/EBPβ.

### 3.11. Suppressing the Fugitive: Arresting Cell Growth through Targeted Kinase Inhibition by Novel Indolocarbazoles

Kevin D. O’Shea * and Florence O. McCarthy

Department of Chemistry and ABCRF, University College Cork, Western Road, Cork, Ireland

* Author to whom correspondence should be addressed; E-Mail: kevin_oshea@umail.ucc.ie.

Altered activity of protein kinases is associated with numerous disease states, including cancer. Their targeted inhibition is a subject of much research (Cohen, P., *et al*. *Nat. Rev. Drug Discov.* 2002, *1*, 309). Many analogues of staurosporine (**1**), a potent but non-specific lead, have been synthesised in order to advance kinase inhibition potential (Peifer, C., *et al*. *J. Med. Chem.* 2006, *49*, 1271). The importance of incorporating a 7-azaindole nucleus in the core framework has recently been accentuated within our group with **2** showing particular potency across a 60-cell screen (Cahill, M.C., *et al*. Ph. D. Thesis, National University of Ireland, Cork, 2013). Incorporation of novel aryl components (e.g., phenyl, 3,4,5-trimethoxyphenyl, thiophenyl) within the frame is also of particular interest with the potential to uncover novel binding mechanisms within the kinase active site. The core aims of this project, therefore, are the design and synthesis of novel kinase inhibitory compounds through modification of structure 3. Synthetic presented here work focuses on the incorporation of a 7-azaindole(or indole) nucleus within the indolocarbazoles frame (either as a bisindolyl or aryl system), utilising a hydroxymaleimide as a replacement for the lactam/maleimide and forming a series of novel derivatives through substitution on the indole nitrogens. Biological evaluation via the NCI 60 cell line screen has been completed for all final compounds thus far with some showing particular activity towards leukaemia, breast cancer, renal cancer and melanoma cell lines.

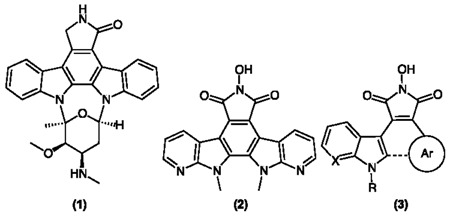


### 3.12. Determination of Log D Value by Micro Shake Flask and LC/MS

Fabian Galla, Jörg Fabian, Bernhard Wünsch *

Institute of Pharmaceutical and Medicinal Chemistry, Westfälische Wilhelms-Universität Münster, Corrensstrasse 48, 48143, Germany

* Author to whom correspondence should be addressed; E-Mail: wuensch@uni-muenster.de.

Lipophilicity is one of the most important physicochemical parameter in Medicinal Chemistry and should be evaluated as early as possible. It can be expressed as the logarithmic distribution of a compound between n-octanol and an aqueous phase, called log D value. It is correlated to other important properties of a compound like protein binding, metabolism or membrane permeability (Hartmann, T., *et al*. *Drug Discov. Today: Technol.* 2004, *1*, 431–439). Additionally the relationship between the log D value and solubility exists (Hill, A.P., *et al*. *Drug Discov. Today* 2010, *15*, 648–655). This is important, because nearly every assay is performed in an aqueous media. Beside different liquid chromatography methods (gradient or isocratic) and the slow stirring methods, the shake flask method is accepted as gold standard for determination of the log D value (*OECD Guidelines*; pp. 107, 117, 123). The talk will present the procedure and advantages of the method, which are as following: low compound consumption, the use of DMSO stock solutions which were also utilized in many assay protocols and the compounds of interest do not need to carry chromophores. The method is very versatile because aqueous, organic or both phases can be quantified. Furthermore the pH value of the aqueous layer can be adjusted to get information about a certain environment. Beside the log D value of test set of compounds (approved drugs), the log D value of novel matrix-metallo proteinase inhibitors (MMPI) have been successfully evaluated. The results of log D value determination by micro shake flask and following LC/MS quantification of these novel MMPIs were in accordance with values obtained from micro shake flask methods of ^18^F-radiolabeled analogues of the compounds.

## 4. Posters

### 4.1. Structure-Activity Relationship Studies on 1-Indazol-1-ylpropan-2-ones as Inhibitors of cPLA2α

Jan Althaus, Walburga Hanekamp and Matthias Lehr *

Institute of Pharmaceutical and Medicinal Chemistry, University of Münster, Corrensstrasse 48, Münster 48149, Germany

* Author to whom correspondence should be addressed; E-Mail: lehrm@uni-muenster.de.

Cytosolic phospholipase A_2_α (cPLA_2_α) plays an important role in inflammatory responses as well as in the progression of tumors. cPLA_2_α selectively cleaves the sn-2 position of arachidonoyl-glycerophospholipids of biomembranes to generate free arachidonic acid and lysophospholipids. Arachidonic acid can be converted to prostaglandins and leukotrienes. Prostaglandin E_2_ is a pro-angiogenic mediator that promotes the growth of tumor blood vessels. Furthermore, certain lysophospholipids lead to resistance of tumors to radiotherapy by increasing the viability of tumor cells. An overexpression of cPLA_2_α was observed in many tumors like non-small-cell lung cancer, cholangiosarcomas, oesophageal cancers and cancers of the colon and small intestine (Linkous, A., *et al*. *Cell. Microbiol.* 2010, *12*, 1369–1377). Thus, inhibitors of this enzyme may represent novel anti-cancer agents.

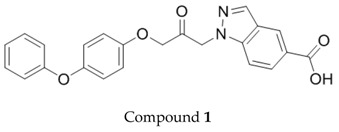


Recently, we have found that indazol-5-carboxylic acid **1** is a potent inhibitor of cPLA_2_α. Since the carboxylic acid functionality of this compound is easily glucuronidated, it could be replaced by other groups such as sulfonamides, amides, ureas and carbamates to increase the metabolic stability. The results of these structural variations are presented.

### 4.2. Structure-Activity Relationship Studies on 1-heteroarylpropan-2-ones as Inhibitors of FAAH

David Garzinsky ^1^, Walburga Hanekamp ^1^, Oliver Koch ^2^ and Matthias Lehr ^1,^*

^1^ Institute of Pharmaceutical and Medicinal Chemistry, University of Münster, Corrensstrasse 48, Münster 48149, Germany

^2^ Department of Chemistry and Chemical Biology, Technische Universität Dortmund, Otto-Hahn-Straße 6, Dortmund 44227, Germany

* Author to whom correspondence should be addressed; E-Mail: lehrm@uni-muenster.de.

Anandamide is one main endocannabinoid in the mammalian organism. It is formed “on demand” during several pathological disorders and mediates analgesic and anti-inflammatory effects by activation of the cannabinoid receptors CB1 and CB2 (Ahn, K., *et al.* *Expert Opin. Drug Discov.* 2009, *4*, 763–784). Furthermore, anandamide was found to possess anti-tumorigenic properties and apart from its anti-proliferative and pro-apoptotic action, it may affect tumor cell angiogenesis and metastasization (Ravi, J., *et al.* *Oncotarget* 2014, *9*, 2475–2486; Freimuth, N., *et al.* *J. Pharm. Exp. Ther.* 2010, *332*, 336–344). An important enzyme in the endocannabinoid metabolism is fatty acid amide hydrolase (FAAH), which rapidly cleaves the lipid mediator anandamide into arachidonic acid and ethanolamine. Inhibitors of FAAH may provide novel options for the treatment of pain, inflammation and cancer, since inhibition of FAAH prolongs and enhances the action of anandamide.
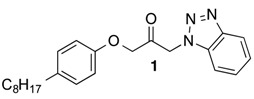


Recently, we have found 1-(1*H*-benzotriazol-1-yl)propanone **1** as a potent inhibitor of FAAH (Forster, L., *et al.* *Bioorg. Med. Chem.* 2010, *18*, 945–952). For the pronounced inhibitory potency of this compound the nitrogen atoms in position 2 and 3 of the benzotriazole scaffold were of special importance. To get more insights into structure-activity relationships, nitrogen atoms were introduced into the annulated benzene ring of the benzotriazole **1**, and the corresponding indole, indazole and benzimidazole derivatives were synthesized. The results of these structural variations are presented together with computational docking studies, describing the interactions between these inhibitors and the active site of FAAH.

### 4.3. Development of New Anthracen-9,10-Dione Derivatives as an Inhibitor of Human Protein Kinase ck2

Samer Haidar *, Annika Meyers, Andre Bollacke and Joachim Jose

Institut für Pharmazeutische und Medizinische Chemie, PharmaCampus, Westfälische Wilhelms-Universität Münster, Corrensstr. 48, Münster 48149, Germany

* Author to whom correspondence should be addressed; E-Mail: shaid_01@uni-muenster.de.

Protein kinase CK2 is an emerging target for the therapeutic intervention in human diseases, in particular in cancer. Inhibitors of this enzyme are at current in clinical trials indicating its druggability (Trembley, J.H*.*, *et al*. *BioFactors (Oxf. Engl.)* 2010, *36*, 187–195). Here we report on the synthesis of two derivatives of 2,6-diaryl-anthracene-9,10-dione, one of which, 2,6-di(furan-3-yl)anthracene-9,10-dione compound (**3**) turned out to be active towards CK2, and ATP competitive with an IC_50_ value of 2.35 µM and a K_i_ value of 1.26 µM. Molecular modeling studies were performed using the Molecular Operating Environment (MOE) (CCGI, Montreal, QC, Canada, 2010) to explain the binding affinity of compound **3** in comparison to emodin. These indicated that unlike emodin, compound **3** was not able to form a hydrogen bond with Lys68, although the compound fits well in the active site of human CK2α, which explains the difference in the measured affinity between those two compounds.

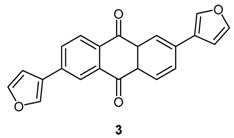


### 4.4. Synthesis and Pharmaceutical Evaluation of Novel Enantiomeric Bicyclic σ_1_ Receptor Ligands

Christian Heselmeyer, Frauke Weber, Bastian Frehland and Bernhard Wünsch *

Institut für Pharmazeutische und Medizinische Chemie, Corrensstraße-48, Münster 48149, Germany

* Author to whom correspondence should be addressed; E-Mail: wuensch@uni-muenster.de.

The σ_1_ receptor is a membrane located protein physiologically expressed in cells of the brain, heart, liver and eyes (Weissmann, A.D., *et al*. *J. Pharmacol. Exp. Ther.* 1988, *247*, 29–33; Mash, D.C., *et al.* *Synapse* 1992, *12*, 195–205; Samovilova, N.N., *et al*. *Eur. J. Pharmacol.* 1988, *147*, 259–264; Ola, M.S., *et al*. *Mol. Brain Res.* 2001, 95, 86–95; Ela, C., *et al*. *J. Pharmacol. Exp. Ther.* 1994, *269*, 1300–1309) and is overexpressed in tumour cells (Vilner, B.J. *Cancer Res.* 1995, *55*, 408–413). In the endoplasmic reticulum the σ_1_ receptor is connected to K^+^ and Ca^2+^ channels (Lupardus, P.D., *et al*. *J. Physiol.* 2000, *526*, 527–539; Hong, W., *et al*. *Eur. J. Pharmacol.* 2000, *408*, 117–125; Vilner, B.J., *et al*. *J. Pharm. Exp. Ther.* 2000, *292*, 900–911). 2,5-diazabicyclo[2.2.2]octane derivatives show higher affinity to human σ_1_ receptors compared to monocyclic piperazine derivatives (Weber, F., PhD dissertation, Westfälische Wilhelms-Universität, Münster, Germany, 2012). Novel bicyclic compounds **1** were designed to reveal high σ_1_ affinity, high selectivity over the σ_2_ subtype and other receptors and high specific cytotoxicity against human tumour cell lines. Herein the synthesis and pharmaceutical evaluation of compounds **1** are presented.

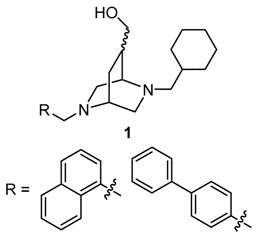


### 4.5. Diverse Effects of Structurally Related Tariquidar-Analogues on Atpase Activity of Abcg2

Maria Karbaum * and Michael Wiese

Pharmaceutical Chemistry II, University of Bonn, An der Immenburg 4, Bonn 53121, Germany

* Author to whom correspondence should be addressed; E-Mail: mkarbaum@uni-bonn.de.

ABCG2 is a membrane protein of the ATP Binding Cassette (ABC) family of proteins. It is expressed in many tissues with barrier or detoxifying functions and is involved in Multidrug Resistance (MDR) in cancer cells. The protein is a primary active transporter using ATP hydrolysis for the extrusion of various chemically unrelated compounds across the plasma membrane (Robey, R.W., *et al*. *Adv. Drug Deliv. Rev.* 2009, *61*, 3–13).

Fluorescent dye based assays are a common tool to identify compounds able to inhibit ABGC2 mediated transport. Furthermore the characterisation of transporter-compound interaction through ATPase activity in membrane vesicles is a well established method (Telbisz, A., *et al*. *Biochim. Biophys. Acta* 2007, *1768*, 2698–2713). Different effects for compounds identified as inhibitors in dye based transport assays regarding the ATPase activity can be observed. Such compounds can show either a stimulating (e.g., quercetin or prazosin) or an inhibitory effect (e.g., vanadat, Ko143 or tariquidar) (Telbisz, A., *et al*. *Eur. J. Pharm. Sci.* 2012, *45*, 101–109).

To facilitate the identification of structural features responsible for these contrary effects on the ATPase activity a series of chemically related compounds derived from tariquidar was studied using both the ATPase assay and functional transport assays with Hoechst 33342 and pheophorbide A as fluorescent dyes. While all of the studied compounds lead to an accumulation of the fluorescent dyes, varying effects on the ATPase activity can be observed, allowing classification of the compounds either as ATPase inhibitors or activators. Compounds containing the complete Tariquidar scaffold consisting of a basic tetrahydroisoquinoline and two amide linked aromatic ring systems inhibit, while structurally corresponding compounds lacking only the tetrahydroisoquinoline moiety activate the ATPase activity. These contrary effects are not correlated with IC_50_ values obtained from dye transport assays.

In addition combination experiments using such analogous pairs of compounds show a competitive interaction suggesting a similar binding mode resulting in opposed effects on the ATPase activity. These findings could allow for a better understanding of different modulation mechanisms of ABCG2 in the future.

### 4.6. Comparison of the Inhibition of Tankyrase-2 by 2-Aryl-7,8-Dihydrothiopyrano[4,3-d]Pyrimidin-4-Ones and 2-Aryl-5,6,7,8-Tetrahydroquinazolin-4-Ones

Michael B.C. Kenny *, Kenneth K.Y. Cheng, Jack Y.C. Cheung, Steven K.M. Fung, Katerina Kumpan, Amit Nathubhai and Michael D. Threadgill

Department of Pharmacy & Pharmacology, University of Bath, Claverton Down, Bath BA2 7AY, UK

* Author to whom correspondence should be addressed; E-Mail: m.kenny@bath.ac.uk.

Tankyrase-1 (PARP-5a, TNKS-1, ARTD5) and tankyrase-2 (PARP-5b, TNKS-2, ARTD6) are members of the poly(ADP-ribose)polymerase (PARP) enzyme superfamily. They currently attract much interest owing to their roles at chromosomal telomeres, at the mitotic spindle and in the wnt signalling pathway, leading to identification as possible targets for drug design for cancer. XAV939 (**1a**), 5-substituted-3-arylisoquinolin-1-ones **2**, 2-arylquinazolin-4-ones **3** and 7-aryl-1-methyl-1,2,3,4-tetra-hydro-1,6-naphthyridin-5-ones **4** have previously been identified as potent and selective inhibitors of the tankyrases (Huang, S.-M.A., *et al*. *Nature* 2009, *461*, 614; Paine, H.A., *et al*. *Bioorg. Med. Chem.* 2015, *in press*; Nathubhai, A., *et al*. *ACS Med. Chem. Lett.* 2013, *4*, 1173; Kumpan, K., *et al*. *Bioorg. Med. Chem.* 2015, *23*, 3013).

As part of an exploration of the structure-activity relationship around the core, short series of 2-aryl-7,8-dihydrothiopyrano[4,3-d]pyrimidin-4-ones **1** and 2-aryl-5,6,7,8-tetrahydroquinazolin-4-ones **5** were synthesised by condensation of the corresponding cyclic β-keto esters with benzamidines. Both series of compounds inhibited tankyrase-2, although the 7,8-dihydrothiopyrano[4,3-d]pyrimidin-4-ones **1** were markedly more potent (IC_50_ 8–38 nM) than the corresponding 5,6,7,8-tetrahydroquinazolin-4-ones **5** (IC_50_ 172–560 nM). A modelling study showed that the two series of compounds bound to the nicotinamide-binding site of the enzyme in conformations with different puckers of the partly saturated rings. Our previous study showed that the tetrahydropyridine ring of **4** does adopt the favoured conformation on binding to tankyrase-2. The inability of the carbocyclic compounds to adop the optimum conformation of the dihydrothiopyrano analogues upon binding may contribute to their lower potency. We thank Worldwide Cancer Research (formerly AICR) for part financial support.

### 4.7. HM30181 Analogs: Influence of Substituents on Inhibiting Potency and Specificity towards Breast Cancer Resistance Protein (bcrp/abcg2)

Sebastian Köhler, Katja Silbermann and Michael Wiese *

Pharmaceutical Chemistry II, University of Bonn, An der Immenburg 4, Bonn D-53121, Germany

* Author to whom correspondence should be addressed; E-Mail: mwiese@uni-bonn.de.

Breast Cancer Resistance Protein (BCRP, ABCG2) belongs to the superfamily of ATP binding-cassette (ABC) proteins. In addition to other physiological functions, it transports potentially cell-damaging compounds out of the cell using the energy from ATP hydrolysis. Certain tumors overexpressing BCRP were found to become resistant against various anticancer drugs.Until now; many efforts have been made to develop useful inhibitors of ABC transporters to improve anticancer therapies. For example, the unspecific inhibitors elacridar (GF120918) and tariquidar (XR9576), and the specific BCRP inhibitors fumitremorgin C (FTC) and its analog Ko143 showed promising results *in vitro*.

We synthesized derivatives of the third-generation P-gp inhibitor HM30181 which is structurally related to tariquidar. In previous work, we investigated the influence of the substitution pattern in the outer phenyl rings on the BCRP modulation. In the present study, we changed substituents in only one phenyl ring to determine their individual impact on inhibition. The compounds were tested for their inhibitory activities against BCRP by Hoechst 33342 and pheophorbide A (PhA) accumulation assays. Enzyme kinetics experiments demonstrated that they are non-competitive inhibitors as they do not affect the binding of the substrate PhA to BCRP. They were screened against P-glycoprotein (P-gp, ABCB1) to confirm the selectivity towards BCRP.The most potent compounds are selective towards BCRP and about 10-fold more potent than the reference XR9577.

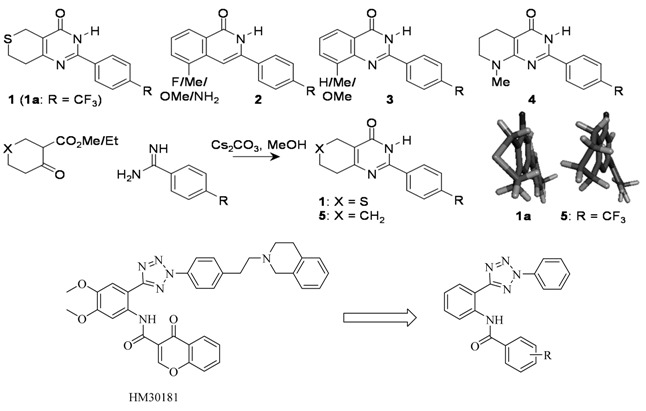


### 4.8. Novel Thiazole Based σ_1_ Receptor Ligands as Potential Anti-Cancer Drugs

Artur Kokornaczyk ^1^ Bernhard Wünsch ^1,^*, Junichiro Yamaguchi ^2^ and Kenichiro Itami ^2^

^1^ Institute of Pharmaceutical and Medicinal Chemistry, Westfälische Wilhelms-Universität Münster, Corrensstrasse 48, Münster 48143, Germany

^2^ Department of Chemistry, Graduate School of Science, Nagoya University, WPI-ITbM, Nagoya University, Furo-cho, Chikusa-ku, Nagoya 464-8602, Japan

* Author to whom correspondence should be addressed; E-Mail: wuensch@uni-muenster.de.

Originally, the opioid receptor was subclassified into three subtypes, which were termed after their prototypical ligands μ (morphine), κ (ketocyclazocine), and σ receptors (SKF-10,047). However, this hypothesis was disproved, since the pharmacological effects of typical σ receptor drugs were not reversed by the opioid receptor antagonists naloxone and naltrexone. Finally, σ receptors were recognized as specific, non-opioid, non-PCP, but haloperidol-sensitive binding sites consisting of σ_1_ and σ_2_ subtypes. The two σ receptor subtypes can be differentiated by molecular weight, tissue distribution, and ligand binding profile. It has been shown that the σ_1_ receptor plays an important role in several socially relevant human diseases including schizophrenia, depressin, Alzheimer’s desease, and drug/alcohol addiction. The discovery of the presence of σ_1_ and σ_2_ receptors in many human tumor cell lines opens up a new area of cancer research (Wojcieh, B.T., *et al*. *Cancer Res.* 1991, 51, 6558–6582; Vilner, B.J., *et al*. *Cancer Res.* 1995, *2*, 408–413). It was found that σ_1_ receptors are expressed in human neoplastic breast epithelial cells (Wang, B., *et al*. *Breast Cancer Res. Treat.* 2004, *3*, 205–214). The σ_1_ receptor antagonists, like haloperidol, rimcazole, and BD-1047 produced a dose-dependent inhibition of the growth of these cells at high concentrations. Due to this overexpression of σ_1_ receptor in serval humane tumor cell lines, the σ_1_ receptor is an interesting target for tumor therapy and diagnosis. However, haloperidol, rimcazole, and BD-1047 are not very selective σ_1_ receptor antagonists. Therefore, novel selective σ_1_ receptor ligands have to be designed and synthesized. The previously synthesized spirocyclic piperidine **1** represent promising σ_1_ receptor ligands. In order to increase the polarity of the rather lipophilic spiro compound **1** the thiazole derivative **2** were designed, synthesized and pharmacologically evaluated. Structure of spirocyclic piperidine **1**, and thiazole piperidine **2**.

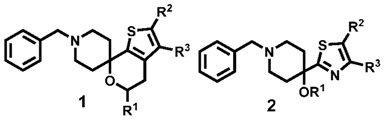


### 4.9. New Potent Inhibitors of Abcg2 Based on the Structure of Gefitinib

Michael Krapf, Jennifer Gallus and Michael Wiese *

Department of Pharmaceutical Chemistry II, University of Bonn, An der Immenburg 4, Bonn 53121, Germany

* Author to whom correspondence should be addressed; E-Mail: mwiese@uni-bonn.de

Since its discovery in 1998, Breast Cancer Resistance Protein (BCRP/ABCG2) stays in the focus of scientific investigations. ABCG2 is a member of the superfamily of ATP binding cassette proteins, which transport a wide variety of different molecules across extra- and intracellular membranes by using the energy from ATP hydrolysis. Unfortunately, many chemotherapeutic agents like mitoxantrone are also transported out of cells by this mechanism, which may lead to a failure of chemotherapeutic cancer treatment. A possible way to overcome this multidrug resistance (MDR) is to inhibit ABCG2 with potent inhibitors (Doyle, A.L., *et al*. *Oncogene* 2003, *22*, 7340–7358; Noguchi, K., *et al*. *Adv. Drug Deliv. Rev.* 2009, *61*, 26–33).

It was found that compounds bearing a quinazoline scaffold, like the tyrosine kinase inhibitor (TKI) gefitinib, were able to inhibit BCRP. In this work different 2-phenylquinazoline derivatives were synthesized to investigate the inhibitory effect on BCRP transport activity. Starting from the 2-phenylquinazoline scaffold, a variety of substituted anilines was introduced at position 4.

The compounds were tested for inhibitory activity against BCRP in the Hoechst 33342 accumulation assay, using MDCK II BCRP and sensitive cells. Additionally, a pheophorbide A accumulation assay was performed for the most potent compounds to compare the obtained results with the Hoechst 33342 assay. Among the tested compounds some showed higher inhibitory activity than Ko143, which is the most potent BCRP inhibitor known till now.

Selectivity of the substances was investigated with a calcein AM accumulation assay. For this purpose P-gp overexpressing A2780adr and MRP1 overexpressing H69 AR cell lines were used. All investigated compounds showed negligible inhibitory effects for both transport proteins.

Cytotoxicity of the compounds was determined with a MTT assay, yielding low toxicities in the higher micromolar range. Furthermore, the most potent substances were investigated for the ability to reverse MDR in MDCK II BCRP cells, using SN-38 as cytotoxic substrate of BCRP. The obtained reversal of the resistance showed, that the active transport of SN-38 out of the cells was effectively inhibited by the compounds.

### 4.10. Angularly Anellated Lapacho Analogues with Antiproliferative Activity against K562 Leukemia Cells

H. Löcken * and K. Müller

Institute of Pharmaceutical and Medicinal Chemistry, University of Münster, Corrensstraße 48, Münster 48149, Germany

* Author to whom correspondence should be addressed; E-Mail: loeckenh@uni-muenster.de.

Recent developments in our group have shown that—besides a series of 2-substituted, linearly anellated naphtho[2,3-*b*]thiophene-4,9-diones **1** with promising activity (Bannwitz, S., *et al*. *J. Med. Chem.* 2014, *57*, 6226–6239)—also some novel C-2-acylated derivatives of the angularly anellated ortho-quinoid analogues **2** exhibit antiproliferative activity against the growth of human keratinocytes.
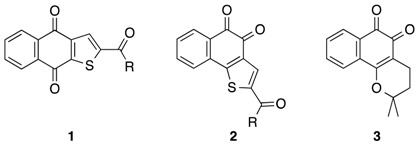


Due to the knowledge of the structurally related *β*-lapachone (**3**) as a potent anti-tumor agent (Gómez Castellanos, J.R., *et al*. *J. Ethnopharmacol.* 2009, *121*, 1–13), we extended our investigations on these structures towards studies on leukemia cells. We have prepared a series of C-2-acylated naphtho[1,2-*b*]thiophene-4,5-diones and studied their antiproliferative activity. The inhibition of the growth of human keratinocytes was determined against HaCaT cells. Using the human chronic myelogenous K562 leukemia cell line for *in vitro* screening, we found that several compounds of this series display promising antiproliferative activities.

### 4.11. Identification of New Plant Derived Inhibitors of Human Hyaluronidase Hyal 1, a Target in Prostate and Bladder Cancer

Isabelle Lengers ^1,^*, Zoya Orlando ^1^, Matthias F. Melzig ^2^, Armin Buschauer ^3^, Andreas Hensel ^4^ and Joachim Jose ^1^

^1^ Institute of Pharmaceutical and Medicinal Chemistry, and d, Institute of Pharmaceutical Biology and Phytochemistry PharmaCampus, Westfälische Wilhelms-Universität, Corrensstraße 48, Münster 48149, Germany

^2^ Institute of Pharmacy, Freie Universität Berlin, Königin-Luise-Str. 2+4, Berlin 14195, Germany

^3^ Institute of Pharmacy, Department of Pharmaceutical/Medicinal Chemistry II, University of Regensburg, Universitätsstr. 31, Regensburg 93040, Germany

^4^ Institut für Pharmazeutische Biologie und Phytochemie, Corrensstr. 48, 48155 Münster, Germany

* Author to whom correspondence should be addressed; E-Mail: isabelle.lengers@uni-muenster.de.

Hyaluronic acid (HA) is a negatively charged polysaccharide comprised of repeating disaccharide units of d-glucuronic acid and *N*-acetyl-d-glucosamine. The physiological and pathophysiological functions of HA depend on its chain size. Space-filling, anti-inflammatory and antiangiogenic effects are triggered by high molecular weight HA (HMW HA) (>20 kDa). Its hydrolysis by hyaluronidases leads to low molecular weight HA (LMW HA) (<20 kDa), resulting in inflammatory and angiogenic effects (Stern, R., *et al*. *Semin. Cancer Biol.* 2008, *18*, 275–280). The degradation of HA is mainly catalyzed by human hyaluronidase Hyal-1. It has been shown that the expression level of Hyal-1 is elevated in cancer cells, like prostate or bladder tumour cells (Lokeshwar, V.B., *et al*. *J. Urol.* 2000, *163*, 348–356; Lokeshwar, V.B., *et al*. *J. Biol. Chem.* 2001, *276*, 11922–11932). For this reason Hyal-1 is an interesting target for drug discovery. The surface display of active Hyal-1 on *Escherichia coli*, via Autodisplay, enables the screening for potential inhibitors in a whole cell system. Based on this technique we determined the inhibitory effect of different plant-extracts on human Hyal-1. The IC_50_ values of *Malvae sylvestris flos*, *Equiseti herba* and *Ononidis radix* were determined to lay between 1.4 and 1.7 mg/mL. Furthermore, the IC_50_ values of four saponines were determined. The obtained IC_50_ value for glycyrrhizinic acid, a known Hyal-1 inhibitor, was 177 µM. The IC_50_ values for the newly identified inhibitors gypsophila saponin 2, SA1641, and SA1657 were 108 µM, 296 µM and 371 µM, respectively. These extracts and compounds identified can be used as a starting point for the synthesis of new small molecule inhibitors targeting human Hyal-1.

### 4.12. Design, Synthesis, and Evaluation of the Azetidin-2-Ones as Novel Bioactive Compounds

Azizah Malebari * and Mary J. Meegan

School of Pharmacy & Pharmaceutical Sciences, Trinity Biomedical Sciences Institute, Trinity College Dublin, Dublin 2, Ireland

* Author to whom correspondence should be addressed; E-Mail: melibaa@tcd.ie.

The African willow tree *Combretum caffrum* Kuntze (Combretaceae) is a very productive source of cancer cell growth (murine P388 lymphocytic leukemia) inhibitory stilbenes, bibenzyls, and phenanthrenes. The synthesis of a series of rigid analogues of combretastatin A-4 is described which contain the four membered β-lactam heterocyclic azetidinone in place of the usual ethylene bridge present in the natural combretastatin stilbene products (O’Boyle, N.M., *et al*. *J. Med. Chem.* 2010, *53*, 8569–8584). The structure-activity relationships of antiproliferative β-lactams, focusing on modifications at the 3- and 4- position of the β-lactam ring, are described.

Synthesis of this series of compounds was achieved utilizing the Staudinger and Reformatsky reactions. To investigate the importance of the OCH_3_ substituent on C-4 of the aryl Ring B moiety for the biochemical activity and potency, a series of thioether S-CH_3_ and S-CH_2_-CH_3_ to replace the oxygen substituent at the C-4 position of the Ring B aryl moiety were synthesized. The compounds which were synthesized have been characterized by ^1^H-NMR and ^13^C-NMR spectroscopy, IR spectroscopy and high resolution mass spectroscopy and X-Ray analysis.

Of a diverse range of heterocyclic derivatives, 3-vinyl and 3-hydroxy analogues displayed the highest potency in human MCF-7 breast cancer cells with IC_50_ values of 9–20 nM, comparable to combretastatin A-4. Further studies are in progress to further rationalize SAR for this series of azetidinones and to confirm that their molecular target is the tubulin and to determine the antiangiogenic effects of these compounds.

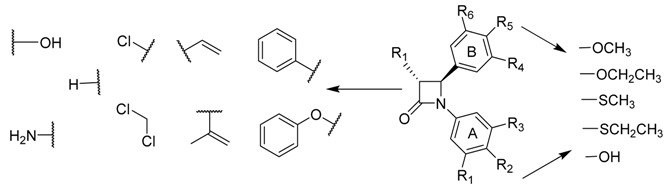


### 4.13. Isoellipticine: Targeting Cell Proliferation by a Structured Approach

Mary McKee * and Florence O. McCarthy

Department of Chemistry, Analytical and Biological Chemistry Research Facility, University College Cork, Western Road, Cork, Ireland

* Author to whom correspondence should be addressed; E-Mail: f.mccarthy@ucc.ie

Ellipticine is a nautral product which possesses multimodal anticancer activity. Some of its modes of action include DNA intercalation, topoisomerase II inhibition, p53 modulation and formation of cytotoxic adducts. While ellipticine itself is not suitable for therapeutic use, two derivatives, 2-methyl-9-hydroxyellipticinium acetate and 2-(2-(diethylamino)ethyl)-9-hydroxyellipticiunium chloride have progressed to clinical trials. Despite this, the production of analogues which have increased selectivity and potency remains a significant challenge (Acton, E.M., *et al*. *J. Med. Chem.* 1994, *37*, 2185; Auclair, C., *et al*. *Cancer Research* 1987, *47*, 6254; Clarysse, A., *et al*. *Eur. J. Cancer* 1984, *20*, 243).

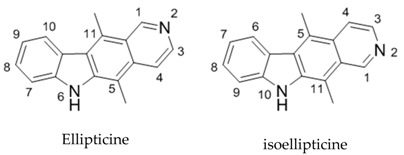


The introduction of substituents at the N6 and C9 position have been shown to increase the cytotoxic activity of the molecule. Therefore we have undertaken substitution of an isomer of ellipticine, isoellipticine with particular emphasis on analogues substituted at the C7 and N10 positions. Initially, the synthesis of isoellipticine was carried out using a route divised by Gribble *et al*., with novel routes explored as a means to increase the yield (Saulnier, M.G., *et al*. *J. Org. Chem.* 1983, *48*, 2690). While N2 substituted analogues have been shown to have varying levels bioactivity, further modification at the N10 and C7 positions has been poorly explored (Russell, E.G., *et al*. *Invest. New Drugs* 2014, *32*, 1113). We have identified that 7-substitutued isoellipticines can cause G2/M arrest in MV4-11 cells, Molt-3, K563 and HL60 among others (Miller, C.M., *et al*. *Org. Biomol. Chem.* 2012, *10*, 7912). We will present the details of the synthesis of isoellipticine derivatives as well as their anticancer activity.

### 4.14. Improved Inhibitors of Human Kinase Protein CK2: Synthesis and Biological Evaluation of Substituted Indeno[1,2-b]indole Derivatives

Abdelhamid Nacereddine ^1,2^, Zouhair Bouaziz ^1^, Christelle Marminon ^1^, Marc Le Borgne ^1^ and Joachim Jose ^2,^*

^1^ EA 446 B2C, ISPB, Université Claude Bernard Lyon 1, 8 avenue Rockefeller, Lyon 69373, France

^2^ Institut für Pharmazeutische und Medizinische Chemie, PharmaCampus, Westfälische Wilhelms-Universität Münster, Corrensstraße 48, Münster 48149, Germany

* Author to whom correspondence should be addressed; E-Mail: joachim.jose@uni-muenster.de

The protein kinase CK2 (previously known as casein kinase II) is a ubiquitous, essential and highly pleiotropic serine/threonine kinase with hundreds of endogenous substrates that are implicated in a wide variety of different cellular functions (mainly growth, proliferation, differentiation, and apoptosis).

CK2 is generally described as a tetramer formed of two catalytic (α and α’) and two regulatory subunits (β). CK2 plays a key role in several physiological and pathological processes and has been connected to many inflammatory, autoimmune, infectious disorders and many different human cancer types (prostate, breast, pancreas, hepatocellular carcinoma and chronic lymphocytic leukemia among others).

Indeno[1,2-*b*]indole derivatives have been identified as ATP-competitive inhibitors which are able to fit into the nucleotide-binding pocket of CK2α and displace the ATP (Alchab, F., *et al*. *Pharmaceuticals* 2015, *8*, 279–302; Gozzi, G.J., *et al*. *J. Med. Chem.* 2015, *58*, 265–277).

The aim of the present study was the synthesis of new indeno[1,2-*b*]indole derivatives by alkylation of the phenolic ring (e.g., R = methyl, ethyl, prenyl). This resulted in a series of new compounds which were tested on their biological activity towards human protein kinase CK2. It turned out that the best compounds had IC_50_ in the nano-molar range.

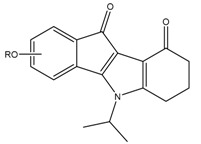


### 4.15. Synthetic Approaches and Biological Evaluation of Combretastatin A-4 Derivatives: New Cis and Iso sulfur Containing Analogs

Abdelfattah Faouzi ^1^, Thierry Lomberget ^1^, Cong Viet Do ^1^, Caroline Barette ^2^, Thi Thanh Binh Nguyen ^1^, Marie-Odile Fauvarque ^2^, Evelyne Colomb ^3^, Marek Haftek ^3^ and Roland Barret ^1^

^1^ EA 4446 B2C, ISPB, Université de Claude Bernard Lyon 1, 8 avenue Rockefeller, Lyon 69373, France

^2^ iRTSV - LBGE - Gen&Chem- Centre de Criblage des Molécules Bio-Actives- CMBA U1038 INSERM/CEA/UJFCEA Grenoble17, rue des Martyrs, 38054 Grenoble Cedex 09, France

^3^ Université Lyon 1, Faculté de Médecine et de Pharmacie, EA 4169 Fundamental, clinical and therapeutic aspects of the skin barrier, 8 avenue Rockefeller, F-69373 Lyon cedex 08, France

* Author to whom correspondence should be addressed; E-Mail: thierry.lomberget@univ-lyon1.fr.

Isolated from the bark of the South African willow tree in the late 1980s by the Pettit research group, combretastatin A-4 or CA-4 is a striking example of tubulin-targeting agents that has displayed a strong inhibitory activity on tumor cells (Pettit, G.R., *et al*. *Experientia* 1989, *45*, 209–211). This product demonstrated to be efficient against a large variety of cancer such as breast, colon, lung or ovarian cancers.

New CA-4 analogs composed of sulfur-containing heterocycles instead of the hydroxymethoxy substituted ring were prepared and evaluated for their *in cellulo* tubulin polymerization inhibition (Vassal, E., *et al*. *J. Biomol. Screen.* 2006, *11*, 377–389) and antiproliferative activities. Therefore, the position of the attachment, the nature of the heterocycle and the *cis* or *iso* bond influences were attested. The presence of a 2-substituted *cis*-benzo[*b*]thiophene moiety grafted to the A trimethoxybenzene exhibited the most promising results and underlined the critical importance of those features in our study (Nguyen, T.T.B., *et al*. *Bioorg. Med. Chem. Lett.* 2012, *22*, 7227–7231).

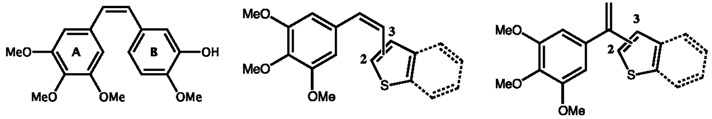


### 4.16. Heterocycle Derived Phenylpiperazinylmethanones as Potent Tubulin Polymerization Inhibitiors

Prinz H ^1,^*, Böhm KJ ^2^, Ridder AK ^1^ and Müller K ^1^

^1^ Institute of Pharmaceutical and Medicinal Chemistry, University of Muenster, Corrensstraße 48, Muenster D-48149, Germany

^2^ Leibniz Institute for Age Research–Fritz Lipmann Institute (FLI), Beutenbergstrasse 11, Jena D-07745, Germany

* Author to whom correspondence should be addressed; E-Mail: prinzh@uni-muenster.de.

We recently described a series of *N*-benzoylated phenoxazines and phenothiazines as potent antitubulin agents. Herein—as a further development in the course of SAR studies - the synthesis and biological evaluation of heterocycle derived phenylpiperazinylmethanones are reported. The *in vitro* evaluation of the antitumor activity and effects on the cell cycle were studied on the human chronic myelogenous K562 leukemia cell line. The most active compounds displayed excellent antiproliferative potencies. To gain further insight into the mode of action, particular analogs were assayed for their effects on cell cycle using a K562 (human chronic myelogenous leukemia) cell-based assay system. Concentrations were determined for 50% cells arrested in G2/M after treatment with the most active compounds.

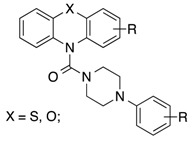


To investigate whether the antiproliferative activities of the novel compounds were related to an interaction with tubulin, selected inhibitors were assayed for their effects on tubulin polymerization using assay conditions as previously described. Several analogs strongly inhibited tubulin polymerization with activities higher or comparable to those of the reference compounds. In conclusion, we have prepared piperazinylmethanones derived from diverse N-heterocycles and have found several of them to be promising and very potent inhibitors of tumor cell growth and tubulin polymerization. The results obtained demonstrate that the antiproliferative potency is related to the inhibition of tubulin polymerization.

### 4.17. Inhibition of CK2: One Target, Two Strategies

Claudia Schmithals, Lukas Kröger, Andre Bollacke, Joachim Jose and Bernhard Wünsch *

Institut für Pharmazeutische und Medizinische Chemie, Westfälische Wilhelms-Universität Münster, Corrensstr. 48, Münster 48149, Germany

* Author to whom correspondence should be addressed; E-Mail: claudia.schmithals@uni-muenster.de.

Protein kinases are enzymes, which are responsible for phosphorylation of proteins. They transfer a phosphate group from ATP to serine/threonine or tyrosine residues. More than 500 protein kinases are known (Litchfield, D.W. *Biochem. J.* 2003, *369*, 1–15; Manning, G., *et al.* *Science* 2002, *298*, 1912–1934).

CK2 (former named as “casein kinase II”) is a special kinase, which phosphorylates both, serine/threonine and tyrosine moieties. CK2 is a heterotetrameric protein consisting of two catalytic subunits (CK2α/CK2α’) and two subunits with regulatory functions (CK2β) (Filhol, O., *et al.* *EMBO Rep.* 2004, *5*, 351–355). In addition to ATP, GTP can also be used as phosphate donor. Further characteristics are the constituent activity and the ubiquitous distribution of CK2. Since CK2 is essential for cell survival, inhibition of CK2 results in apoptosis (Litchfield, D.W. *Biochem. J.* 2003, *369*, 1–15). In various diseases, including cancer, CK2 is upregulated (Guerra, B., *et al.* *Curr. Med. Chem.* 2008, *15*, 1870–1886), indicating that CK2 is an interesting target for the development of new drugs.

Since CK2 is a kinase, the binding of its cosubstrate ATP (or GTP) is mandatory for the catalytic activity. Therefore, it is possible to strongly decrease the activity of the enzyme by ATP site-directed inhibitors (Cozza, G., *et al.* *Expert. Opin. Ther.* 2012, *22*, 1081–1097). The goal of our research is the development of novel potent and selective CK2 inhibitors with dibenzofuran scaffold.

Furthermore there is the possibility to manipulate the function of CK2 by antagonizing the CK2 subunit interaction. While the monomeric catalytic subunits possess a constitutive activity themselves, the formation of the holoenzyme strongly affects the catalytic activity and changes the substrate preference (Laudet, B., *et al.* *Mol. Cell. Biochem.* 2008, *316*, 63–69). Therefore, we are also investigating the syntheses and biological activity of low molecular weight inhibitors of CK2 subunit interaction.

### 4.18. Tumor Cell Targeting with Escherichia coli Displaying Anti-EGFR Antibody Fragments

Wilhelmine Weckenbrock ^1^, Felix Blaßhofer, Lars-Oliver Klotz ^2^, Joachim Jose ^1,*^

^1^ Institute for Pharmaceutical and Medicinal Chemistry, PharmaCampus, Westfälische Wilhelms-University, Corrensstraße 48, Münster 48149, Germany

^2^ Centre for Pharmacy & Health Research, University of Alberta, 11361-87 Avenue, Edmonton, AB T6G 2E1, Canada

* Author to whom correspondence should be addressed; E-Mail: joachim.jose@uni-muenster.de

Bacterial tumor cell targeting describes the use of selective affinity of bacterial cells towards tumorous tissue for cancer diagnosis or treatment (Lee, C.-H. *Appl. Microbiol. Biotechnol.* 2012, *93*, 517–523). The oral or rectal application of bacteria is possible, because lipopolysaccharide (LPS) induced toxicity only applies for systemic use. Therefore, in gastrointestinal cancer, bacteria with distinct affinity for malignant cells could serve as drug carriers delivering a drug precisely to the spot of interest. In case of peptidic drugs, the bacterial cell could serve as both the carrier and the producing unit. The design of tumor targeting bacteria which express a prodrug converting enzyme is also an interesting idea. Thus, a prodrug could be converted into the active agent selectively within malignant tissue.

The epidermal growth factor receptor (EGFR) is a receptor tyrosine kinase (Herbst, R.S. *Int. J. Radiat. Oncol. Biol. Phys.* 2004, *59*, 21–26). EGFR is the target of several drugs, including both small molecule (e.g., gefitinib or erlotinib) and monoclonal antibodies (cetuximab or panitumumab), because the overexpression of EGFR is associated with colorectal tumors and other sorts of cancer (Kamath, S., *et al.* *Med. Res. Rev.* 2006, *26*, 569–594). Due to its location on the tumors cell surface EGFR is a suitable target for bacterial drug targeting.

The aim of this study was to design a strain of *E. coli* with distinct affinity towards EGFR. For this purpose, anti-EGFR single chain variable fragment (scF_v_) 425 (Horn, U., *et al.* *Appl. Microbiol. Biotechnol.* 1996, *46*, 524–532) was displayed on the surface of *E. coli* using the AIDA-I-autotransporter technology (Jose, J., *et al.* *Microbiol. Mol. Biol. Rev.* 2007, *71*, 600–619). The affinity of *E. coli* cells displaying the antibody fragment towards tumor cells overexpressing EGFR was demonstrated in FACS and fluorescence microscopic assays. Specificity of binding to the EGF-receptor was proven in experiments using siRNA down-regulation of this receptor. Our findings demonstrate that *E. coli* displaying antibody fragments can be used for tumor cell targeting *in vitro*. Further experiments on the applicability of this technology *in vivo* are needed.

### 4.19. Using Proximity-Dependent Biotinylation (Bio-ID) to Detect Novel Interaction Partners of the Human Pdcd4 Protein

Sandeep Dukare * and Karl-Heinz Klempnauer

Institut für Biochemie, Westfälische-Wilhelms-Universitat, Münster 48149, Germany

* Author to whom correspondence should be addressed; E-Mail: s_duka01@uni-muenster.de.

Proximity-dependent biotinyation is a new method for the identification of interaction partners and proximal proteins of a protein of interest (Roux, K.J., *et al*. *J. Cell Biol.* 2012, *196*, 801–810). We have used this method to search for novel interaction partners of the programmed cell death 4 (Pdcd4) tumor suppressor protein. Pdcd4 is a nuclear-cytoplasmic shuttling protein whose expression is down-regulated in various tumors. Pdcd4 is known to play a role in different cellular processes, such as transcription and translation by interacting with various cellular proteins. Here, we report the identification of various novel interactions of human Pdcd4 as detected by mass spectrometry.

### 4.20. Tau Aggregation Inhibitors in Alzheimer’s Disease

Marie Jouanne *, Sébastien Coufourier, Victor Babin, Sylvain Rault and Anne-Sophie Voisin-Chiret

Université de Caen Normandie, CERMN (Centre d’Etudes et de Recherche sur le Médicament de Normandie UPRES EA 4258-FR CNRS 3038 INC3M, Bd Becquerel), Caen F-14032, France

* Author to whom correspondence should be addressed; E-Mail: marie.jouanne@unicaen.fr.

Alzheimer’s disease a progressive neurodegenerative disorder, is the leading cause of dementia in late adult life. Pathologically, it is characterized by intracellular neurofibrillary tangles (NFTs) and extracellular amyloidal protein deposits contributing to senile plaques. In this poster, we focus on NFTs composed largely of paired helical filaments (PHFs) with hyperphosphorylated tau proteins. Tau protein aggregation is a multifactorial process (Buee, L., *et al*. *Brain Res. Rev.* 2000, *33*, 95–130) which allows to consider different inhibitors.

Tau aggregation, a multifactorial process; Targets for potential inhibitors (Guzmán-Martinez, L., *et al*. *Fneur* 2013, *4*, 1–6).

Among all aggregation factors, we will highlight on misfolding phenomena (Ramirez-Alvaro, M., *et al*. *Protein Misfolding Diseases: Current and Emerging Principles and Therapies*, 2010, Wiley, Hoboken, NJ, USA) and consider possible inhibitors such as abiotic foldamers.

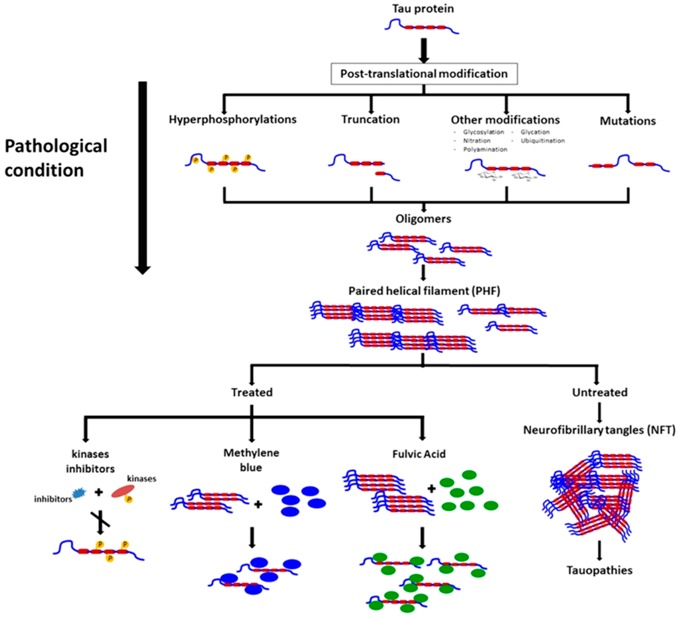


### 4.21. First Evidence that Oligopyridines, Alpha Helix Foldamers, Inhibit Mcl-1 and Sensitize Ovarian Carcinoma Cells to Bcl-x_L_-Targeting Strategies

Charline Kieffer ^1,2^, Siham Hedir ^1,3^, Jana Sopkova-de Oliveira Santos ^1,2^, Philippe Juin ^4,5^, Sylvain Rault ^1,2^, Laurent Poulain ^1,3^ and Anne Sophie Voisin-Chiret ^1,2,^*

^1^ Normandie University, 14032 Caen Cedex 5, France

^2^ UNICAEN, CERMN-FR CNRS INC3M, 14032 Caen postal code, France

^3^ UNICAEN, BioTICLA UMR 1199 INSERM, Caen, France; Comprehensive Cancer Center François Baclesse, 14032 Caen postal code, France

^4^ Team 8, Nantes-Angers Centre for Cancer Research, Unité INSERM 892 - CNRS 6299 Centre de Recherche en Cancérologie Nantes-Angers. 44077 Nantes-Cedex, France

^5^ Institut de Cancérologie de l’Ouest, Comprehensive Cancer Center R. Gauducheau, 44805 Nantes, France.

* Author to whom correspondence should be addressed; E-Mail: anne-sophie.voisin@unicaen.fr.

Apoptosis control defect such as the deregulation of Bcl-2 family members expression is frequently involved in chemoresistance. In ovarian carcinoma, we previously demonstrated that Bcl-x_L_ and Mcl-1 cooperate to protect tumor cells against apoptosis and their concomitant inhibition leads to massive apoptosis even in absence of chemotherapy. Furthermore, Mcl-1 down-regulation or inactivation was required to sensitize cells to Bcl-x_L_-targeting strategies. Whereas Bcl-x_L_ inhibition is accessible (using BH3-mimetics), Mcl-1 inhibition is problematic. In this context, we designed and synthesized oligopyridines potentially targeting Mcl-1 hydrophobic pocket, evaluated their capacity to inhibit Mcl-1 in live cells and implemented functional screening assay to evaluate their ability to sensitize ovarian carcinoma cells to Bcl-x_L_-targeting strategies. We established structure-activity relationships and we focused attention on MR29072, named pyridoclax. Without cytotoxic activity as single agent, pyridoclax inhibits Mcl-1 in combination with Bcl-x_L_-targeting siRNA or with ABT-737 against ovarian, lung cancer cells and in mesothelioma.

### 4.22. Synthesis of Epoxybenzoxocines as Conformationally Restricted Nmda Receptor Antagonists

Samuel Asare-Nkansah * and Bernhard Wünsch

Institut für Pharmazeutische und Medizinische Chemie, Corrensstraße-48, Münster 48149, Germany

* Author to whom correspondence should be addressed; E-Mail: s_asar01@uni-muenster.de.

The *N*-methyl-d-aspartate (NMDA) receptor is a ligand-gated ion channel that plays a prominent role in various central nervous system (CNS) events such as development of neurons, synaptic plasticity, memory and learning (Ono, S., *et al*. *Chem. Pharm. Bull.* *(Tokyo)* 2002, *50*, 966–968). The NMDA receptor is highly permeable to Ca^2+^ ions, and under pathological conditions, elevated intracellular Ca^2+^ ion concentrations up to cytotoxic levels (excitotoxicity) contribute partly to neuronal death (Tech, G. *NIH Public Access* 2011, *51*, 18). The NMDA receptor is consequently implicated in diseases of the CNS (Alzheimer’s disease, Parkinson’s disease, Huntington’s disease and epilepsy), which are associated with excitotoxicity (Flores-Soto, M.E., *et al*. *Neurologia* 2012, *27*, 301–310). Therefore, the NMDA receptor represents a potential therapeutic target for the development of innovative drugs for the treatment of neurological disorders.

The 1,3-dioxolane derivatives dexoxadrol (**1**) and etoxadrol (**2**) have high NMDA receptor affinity with non-tolerable side effects including retrograde amnesia and psychotomimetic effects (Aepkers, M., *et al*. *Bioorg. Med. Chem.* 2005, *13*, 6836–6849). Studies performed on **1** and **2** have led to the development of 1.3-dioxane analogs **3** with even higher affinity towards the phencyclidine (PCP) binding site of the NMDA receptor (Aepkers, M., *et al*. *Bioorg. Med. Chem.* 2005, *13*, 6836–6849; Utech, T., *et al*. *Eur. J. Med. Chem.* 2011, *46*, 2157–2169). The current project examines the influence of conformational restriction of **3** on the NMDA receptor affinity and selectivity. It is envisaged that ligands **4** with a phenyl moiety fixed in axial orientation will give further insights into the requirements for high NMDA receptor affinity.

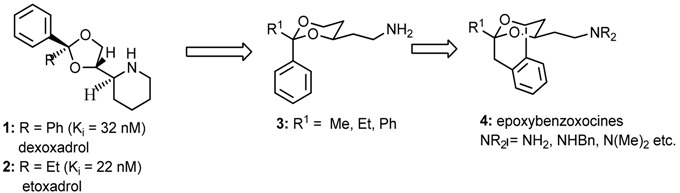


### 4.23. Benzo[7]Annulenes as GluN2B Selective NMDA Receptor Antagonists: Effect of NO_2_ Group on Affinity and Selectivity

Sandeep Gawaskar ^1,2,3,^*, Dirk Schepmann ^1^ and Bernhard Wünsch ^1,2,3^

^1^ Institute für Pharmazeutische und Medizinische Chemie, WWU Münster, Corrensstr. 48, Münster 48149, Germany

^2^ Graduate School of Chemistry, Wilhelm-Klemm-Str. 10, Münster 48149, Germany

^3^ Cells-in-Motion Cluster of Excellence (EXC 1003-CiM) WWU Münster 48149, Germany

* Author to whom correspondence should be addressed; E-Mail: s_gawa01@uni-muenster.de.

The obligatory heterotetrameric NMDA receptor plays a vital role in synaptic plasticity, memory and learning. The receptor can be composed of GluN1a-h (eight splice variants), GluN2A-D or GluN3A-B subunits. However, a functional NMDA receptor requires at least one GluN1 and one GluN2 subunit. The simultaneous binding of agonists glycine and (*S*)-glutamate, at the GluN1 and GluN2 subunits, respectively, leads to the activation of the NMDA receptor and allows passage of cations like Ca^2+^ and Na^+^ into the neuronal cell. Excess glutamate triggers overactivation of NMDA receptors (Paoletti, P., *et al*. *Curr. Opin. Pharmacol.* 2007, *7*, 39). The resultant Ca^2+^ influx leads to neuronal cell death, termed as excitotoxicity, which is considered causal to the progression of neurodegenerative disorders like Alzheimer’s disease and Parkinson’s disease.

Allosteric inhibitors like Ro 25-6981 (**1**) (Fischer, G., *et al*. *J. Pharmacol. Exp. Ther.* 1997, *283*, 1285), binding at the GluN1-GluN2B interface, prevent (*S*)-glutamate from interacting with its orthosteric binding site. This event allows the ion channel to remain closed and inhibits Ca^2+^ influx, resulting in neuroprotective potential. The limited expression of the GluN2B subunit in only some parts of the central nervous system offers better side effect profile to allosteric inhibitors compared to unselective channel blockers.

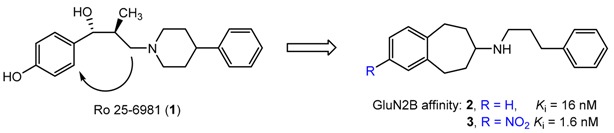


Starting from Ro 25-6981 (1), with the aim of overcoming issues related to selectivity and low oral bioavailability associated with classical GluN2B-targeting allosteric inhibitors, a conformational restriction approach (Gawaskar, S., *et al*. *Bioorg. Med. Chem.* 2014, *22*, 6638) led to the benzo[7]annulenes **2** and **3**. Pharmacological evaluation of amines 2 and 3 demonstrates that the NO_2_ moiety in 2-position and the length of the phenylalkyl residue in 7-position play crucial roles in modulating GluN2B binding affinity. Docking studies showed that the NO_2_ moiety interacts with a water molecule in the binding site, which further forms a network of interactions with backbone amino acid residues.

### 4.24. Synthesis of Substituted Carbazole Derivatives as CB_2_ Receptor PET Tracers

Dominik Heimann and Bernhard Wünsch *

PharmaCampus, Institut für Pharmazeutische und Medizinische Chemie, Westfälische Wilhelms-University Münster, Münster 48149, Germany

* Author to whom correspondence should be addressed; E-Mail: wuensch@uni-muenser.de.

The cannabinoid receptor type 2 (CB_2_) is part of the endocannabinoid system. While it was first considered to be the “peripheral cannabinoid receptor”, it is known today that it also occurs in the central nervous system. There the G protein-coupled receptor is involved in many physiological and pathological processes like Alzheimer’s disease, depression and schizophrenia (Atwood, B.K., *et al*. *Brit. J. Pharmcol.* 2010, *160*, 467–479; Di Marzo, V. *Pharmacol. Res.* 2009, *60*, 77–84). To achieve a better understanding of the regulation of the cannabinoid system in the normal and diseased human brain, non-invasive quantitative visualization of the CB_2_ receptor subtype is highly desirable.

Therefore, highly potent and selective compounds for the central CB_2_ receptor are required, which can be radiolabeled for PET imaging purposes. Compound **1** with the carboxamide substructure possesses high CB_2_ receptor affinity (Ki = 5.8 nM) and selectivity over the CB_1_ receptor (>200). However, it proved to be very lipophilic and metabolically unstable in a CD-1 mouse model. The two major radiometabolites result from enzymatic hydrolysis of the amide bond and the oxidative N-dealkylation of the carbazole nitrogen atom (Teodoro R., *et al*. *Org. Med. Chem. Lett.* **2013**, *3*, 11). Based on these results, a metabolically more stable compound with the carbazole scaffold is planned. Therefore, the bioisosteric replacement of the amide moiety with more stable structural elements like an amine (X = NR) or a ketone (X = C = O) is envisaged.

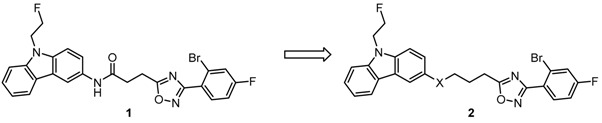


### 4.25. Synthesis of Diastereomerically Pure Penturonic Acid-Derived C-Glycosides and Their Antibacterial Properties

Hannes Müller *, Oriana Agoglitta and Ralph Holl

Institut für Pharmazeutische und Medizinische Chemie, WWU Münster, Corrensstraße 48, Münster 48149, Germany

* Author to whom correspondence should be addressed; E-Mail: h_muel11@wwu.de.

Successful treatment of bacterial infections has become increasingly problematic. Various Gram-negative bacteria (GNB) have developed multi-drug resistance against common antibiotics. Thus, new antibiotics with a novel mode of action are required. A promising new target to treat infections with GNB is LpxC, a Zn^2+^-dependent deacetylase. In GNBLpxC catalyzes the deacetylation of UDP-3-*O*-[(*R*)-3-hydroxymyristoyl]-*N*-acetylglucosamine, which is the first committed step in the biosynthesis of lipid A. Lipid A anchors lipopolysaccharide (LPS) in the outer membrane of GNB. Lack of lipid A and accordingly of LPS leads to the death of most of these bacteria (Barb, A.W., *et al.* *Curr. Pharm. Biotechnol.* 2008, *9*, 9–15).

CHIR-090 has turned out to be a potent inhibitor of LpxC and served as lead-structure for the development of novel LpxC inhibitors (McClerren, A.L., *et al.* *Biochemistry* 2005, *44*, 16574–16583). It consists of a hydroxamic acid moiety to chelate the catalytic Zn^2+^-ion, a hydrophobic region to imitate the acyl-chain of the natural substrate and a linker to mimicits sugar scaffold. In syntheses starting from d-mannose, we synthesized four diastereomers ofpenturonic acid-derived *C*-glycoside **1** with various hydrophobic regions R and tested their antibacterial activity indisk-diffusion assays (Löppenberg, M., *et al.* *Org. Biomol. Chem.* 2013, *11*, 6056–6070).

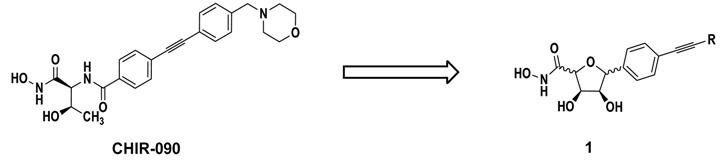


### 4.26. Chiral Pool Synthesis of GluN2B Selective Receptor Antagonists with 3-Benzazepine Scaffold

Susann Rath * and Bernhard Wünsch

PharmaCampus, Institut für Pharmazeutische und Medizinische Chemie, Westfälische Wilhelms-Univ. Münster, Münster 48149, Germany

* Author to whom correspondence should be addressed; E-Mail: s_rath02@uni-muenster.de.

NMDA receptors containing a GluN2B subunit play an important role in neurophysiological processes. For this reason selective GluN2B antagonists are of great interest for the treatment of different neuronal disorders. Ifenprodil was initially synthesized as an α_1_ adrenergic receptor antagonist, but later an even higher affinity to NMDA receptors with the GluN2B subunit was detected (K_i_ = 10 nM, IC_50_ = 13.3 nM). However it proved to be non-selective and led consequently to undesired side effects. Nevertheless ifenprodil served as first lead compound for the design and development of new GluN2B antagonists (Chenard, B.L., *et al*. *J. Med. Chem.* 1991, *34*, 3085–3090; Kemp, J.A., *et al*. *Nat. Neurosci.* 2002, *5*, 1039–1042).

Compounds **1** with the 3-benzazepine scaffold possess high GluN2B affinity and antagonistic activity. One of the most promising compounds is **1a** (R = (CH_2_)_4_Ph) with a K_i_ value of 5.4 nM and an IC_50_ value of 360 Nm (Tewes, B., *et al.* *ChemMedChem* 2010, *5*, 687–695).

Based on these encouraging results, a chiral pool synthesis of diastereo- and enantiomerically pure 3-benzazepine derivatives **2** starting from (*S*)-tyrosine or (*S*)-DOPA is envisaged. According to docking studies 3-benzazepines with a hydroxy moiety in the 8-position should fit nicely into the ifenprodil binding pocket. Additionally the hydroxymethyl moiety in 4-position allows the fine tuning of the GluN2B affinity and, moreover, the introduction of ^18^F to generate a tracer for positron emission tomography (PET).

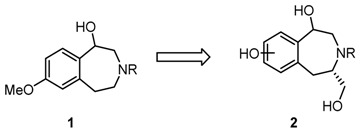


### 4.27. Discovery of Donecopride a Novel Multi-Target Directed Ligands for Alzheimer’s Disease

Christophe Rochais ^1,^*, Cédric Lecoutey ^1^, Thomas Freret ^2^, Céline Ballandonne ^1^, Valentine Bouet ^2^, Patrizia Giannoni ^3^, Florence Gaven ^3^, Sylvie Claeysen ^3^, Michel Boulouard ^2^, Patrick Dallemagne ^1^

^1^ Centre d’Etudes et de Recherche sur le Médicament de Normandie (CERMN)-UPRES EA 4258-FR CNRS INC3M-SFICORE, Université de Caen Basse-Normandie, UFR des Sciences Pharmaceutiques-Bd Becquerel, Caen F-14032, France

^2^ Groupe Mémoire et Plasticité comportementale (GMPc)-A4259-Université de Caen Basse-Normandie, UFR desSciences Pharmaceutiques - Bd Becquerel, Caen F-14032, France

^3^ CNRS, UMR-5203, Institut de Génomique Fonctionnelle, Montpellier F-34000, France

* Author to whom correspondence should be addressed; E-Mail: christophe.rochais@unicaen.fr

Complex pathologies such as Alzheimer’s disease (AD) would benefit from a combination of actions targeting, in the same time, several molecular causes implied in the pathogenesis. Our aim was to design a multi target-directed ligand (MTDL) gathering two properties: acetylcholinesterase (AChE) inhibition and 5-HT4 receptor activation. AChE inhibition is the action mechanism of donepezil, the current available drug for AD. Activation of 5-HT4 receptors promotes the non-amyloidogenic cleavage of the amyloid precursor protein (APP) and the release of the neuroprotective soluble APPalpha fragment (Lecoutey, C., *et al*. *PNAS* 2014, *111*, E3825–E3830).

Combining a dual-binding site pharmacophore of AChE inhibitors with a pharmacophore of 5-HT4R ligands, we isolated a candidate compound and performed pharmacomodulation of this hit to optimize its properties. We selected donecopride1 as a druggable lead able to inhibit AChE (IC50 = 16 nM) and to induce sAPPalpha release (EC_50_ = 11.3nM) upon 5-HT4R activation (Ki = 10.4 nM; 48.3% of serotonin response). *In vivo* properties of this new compound in the 5XFAD mouse model of AD (acute and chronic administration) will be presented.

### 4.28. Structure–Activity Relationship Studies on 1-Heteroaryl-3-Phenoxypropan-2-Ones Acting as Inhibitors of Cytosolic Phospholipase A_2_α and Fatty Acid Amide Hydrolase: Replacement of the Activated Ketone Group by Other Serine Traps

Tom Sundermann *, Walburga Hanekamp and Matthias Lehr

Institute of Pharmaceutical and Medicinal Chemistry, University of Münster, Corrensstrasse 48, Münster 48149, Germany

* Author to whom correspondence should be addressed; E-Mail: tom.sundermann@uni-muenster.de.

Cytosolic phospholipase A_2_α (cPLA_2_α) and fatty acid amide hydrolase (FAAH) are serine hydrolases (Bachovchin, D.A., *et al*. *Nat. Rev. Drug Discov.* 2012, *11*, 52–68). cPLA_2_α is involved in the generation of pro-inflammatory lipid mediators, FAAH terminates the anti-inflammatory effects of endocannabinoids (Dennis, E.A., *et al*. *Chem. Rev.* 2011, *111*, 6130–6185; Di Marzo, V. *Rev. Physiol. Biochem. Pharmacol*. 2008, *160*, 1–24). Therefore, inhibitors of these enzymes may represent new drug candidates for the treatment of inflammation. We have reported that certain 1-heteroarylpropan-2-ones are potent inhibitors of cPLA_2_α and FAAH (Ludwig, J., *et al*. *J. Med. Chem.* 2006, *49*, 2611–2620; Zahov, S., *et al*. *Chem. Med. Chem.* 2011, *6*, 544–549). The serine reactive ketone group of these compounds, which is crucial for enzyme inhibition, is readily metabolized resulting in inactive alcohol derivatives (Fabian, J., *et al*. *Chem. Biol. Interact.* 2013, *206*, 356–363). In order to obtain metabolically more stable inhibitors, we replaced this moiety by α-ketoheterocyle, cyanamide, nitrile and α-ketoamide serine traps. Investigations on activity and metabolic stability of these substances revealed that in all cases an increased metabolic stability was accompanied by a loss of inhibitory potency against cPLA_2_α and FAAH, respectively.

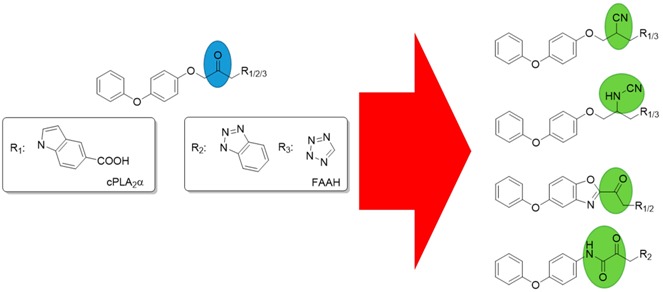


### 4.29. Generation of a Genetically Encoded, Photoactivatable Intein for the Controlled Production of Cyclic Peptides

Jana Böcker * and Henning Mootz

Institute of Biochemistry, University of Münster, Wilhelm-Klemmstr. 2, Münster 48149, Germany

* Author to whom correspondence should be addressed; E-Mail: j_boec09@uni-muenster.de.

Cyclic peptides are important natural products and hold great promise for the identification of new bioactive molecules. They are privileged structures for inhibitor design because their rigidity off-sets an energetic loss of the entropic contribution when binding to a protein target, in comparison to the more flexible linear counterpart. Additional advantages are increased proteolytic stability and membrane permeability. Their large size and protein-like structure make cyclic peptides particularly promising candidates for the inhibition of protein–protein interactions (Arnison, P.G., *et al*. *Nat. Prod. Rep.* 2013, *30*, 108–160). The split-intein-mediated split-intein circular ligation of peptides and proteins (SICLOPPS) technology provides a generic access to fully genetically encoded head-to-tail cyclized peptides (Scott, C.P., *et al*. *Proc. Natl. Acad. Sci. USA* 1999, *96*, 13638–13643). With this method, large libraries of cyclic peptides can be generated *in vivo*. (Lennard, K.R., *et al*. *Chemistry* 2014, *20*, 10608–10614). However, owing to the spontaneous protein splicing reaction, product formation occurs inside cells, making peptide isolation inconvenient and precluding traditional *in vitro* assays for inhibitor discovery. The design of a genetically encoded, light-dependent intein using the photocaged tyrosine derivative *ortho*-nitrobenzyltyrosine (ONBY) incorporated at an internal, non-catalytic position is reported in this study. Stable intein precursors were purified from the *E. coli* expression host and subsequently subjected to light activation *in vitro* for both the regular protein splicing format and cyclic peptide production, including the natural product segetalin H as an example. The activity of the intein could also be triggered in living cells (Böcker, J.K., *et al*. *Angew. Chem. Int. Ed.* 2015, *54*, 2116–2120).

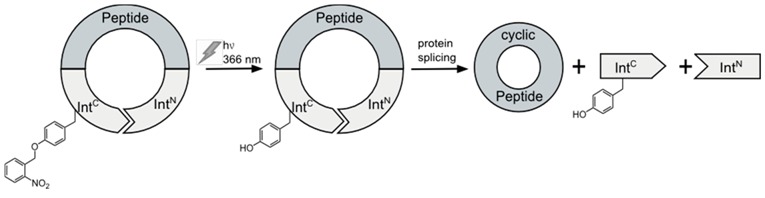


### 4.30. Analysis of Essential Structural Elements by Deconstruction of Benzazepine Based Glun2b Antagonists

Sougata Dey ^1,2,^* and Bernhard Wünsch

^1^ Institut für Pharmazeutische und Medizinische Chemie, Corrensstraße-48, Münster 48149, Germany

^2^ Graduate School of Chemistry, University of Münster, Wilhelm-Klemm-Str.-10, Münster 48149, Germany

* Author to whom correspondence should be addressed; E-Mail: deysou88@gmail.com.

The NMDA receptor has received significant attention due to its unique properties and therapeutic potentials. It is considered as an important therapeutic target for a variety of conditions like epilepsy, ischemia (Di, X., *et al*. *Stroke* 1997, *28*, 2244–2251), depression (Mony, L., *et al*. *Br. J. Pharmacol*. 2009, *157*, 1301–1317) and chronic neurodegenerative diseases like Alzeimer’s disease and Parkinson’s disease (Wessell, R.H., *et al*. *Neuropharmacology* 2004, *47*, 184–194). Without affecting the main pharmacophore, the deconstruction approach of known ligands can provide a clear intuition of essential structural elements. According to this deconstruction-reconstruction approach a lead compound is deconstructed into several fragments and subsequently reconstructed to produce a diverse set of compounds with different functional groups (Chen, H., *et al*. *Drug Discov. Today* 2015, *20*, 105–117).

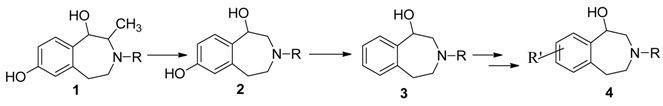


The 3-benzazepine **1** is a conformationally restricted analog of the flexible ligand ifenprodil with high GluN2B affinity (Tewes, B, *et al*. *ChemMedChem* 2010, *5*, 687–695). In this project deconstruction of the lead compound **1** is envisaged, resulting in simplified compounds **3**, which will be decorated with different functional group at the phenyl ring to obtain the compounds **4**. This concept will allow us to identify the essential structural elements for high GluN2B Affinity.

### 4.31. Stereoselective Synthesis of Rhodotorulic acid and Analogues

Timothée Garnerin *, Alexandra Dassonville-Klimpt and Pascal Sonnet

LG2A, CNRS FRE3517, UFR de pharmacie, 1 rue des Louvels, Université de Picardie Jules Verne, 80037Amiens Cedex, France

* Author to whom correspondence should be addressed; E-Mail: timothée.garnerin@etud.u-picardie.fr.

Antibiotic resistance is an emerging disease and a real problem of health. Resistance of Gram negative bacteria such as *Acinetobacter baumannii* and *Escherichia coli* to conventional antibiotic lead to therapeutic failure and require new antibiotherapies. The use of the iron transport systems is one of the most promising strategies to overcome this resistance phenomenon. These specific routes of entry, essential for the survival of the microorganisms, allow ferric siderophore complexes to carry ferrous iron to the bacteria.

These systems can allow the introduction of antibacterial agents (conjugates antibiotic-siderophore) (Budzikiewicz, H. *Curr. Top. Med. Chem*. 2001, *1*, 73–82; Hennard, C., *et al*. *J. Med. Chem*. 2001, *44*, 20139–2151) or toxic complexes (gallium complexes) (Kelson, A.B., *et al*. *Curr. Opin. Pharmacol.* 2013, *13*, 707–716) into the bacteria to kill them. Rhodotorulic acid (RA) is a siderophore recognised by the Fhu receptor expressed by *Acinetobacter baumannii* and *Escherichia coli*. This dioxopiperazine possesses hydroxamate as iron ligands and two asymmetric centers with *S*,*S* configuration. This spatial orientation is essential for the recognition of the iron-siderophore complex by the Fhu receptor.

We have previously reported the asymmetric synthesis of 3-substituted 2-oxopiperazines. We present here an original and a convergent strategy to synthesize RA and 3,5-disubstituted analogues of this siderophore. These compounds will be connected to an antibiotic to test their antibacterial properties and to determine the most efficient according to the nature of the ligands and the absolute configuration of the stereogenic centers.

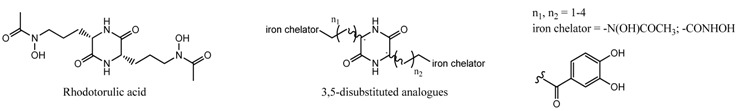


### 4.32. Autodisplayed Lpr1 Iv-Domain as A Tool For Identification of Drug Delivery Agents across the Blood-Brain Barrier

Cristina Gisbert Fenoy ^1,^*, Bastian Raudszus ^2^, Iavor Zlatev ^2^, Christian Nienberg ^1^, Klaus Langer ^2^, Joachim Jose ^1^

^1^ Institute of Pharmaceutical and Medicinal Chemistry, PharmaCampus, WestfälischeWilhelms-UniversitätMünster, Corrensstraße 48, Münster 48149, Germany

^2^ Institute of Pharmaceutical Technology and Biopharmacy, PharmaCampus, WestfälischeWilhelms-UniversitätMünster, Corrensstraße 48, Münster 48149, Germany

* Author to whom correspondence should be addressed; E-Mail: cristina.gisbert@uni-muenster.de.

Low-density lipoprotein receptor-related protein-1 (LPR1) is a widely expressed cell-surface receptor with diverse biological functions, including ApoE binding and endocytosis. LRP1 is abundantly expressed in neurons and the vascular system, making it an efficient target for delivery of therapeutic substances across the blood-brain barrier (BBB), in case these are part of a system, like nanoparticles, whereApoE is present (Wagner, S., *et al*. *PLoS ONE* 2012, *7*, e32568). Previous studies showed the high binding affinity of ApoE to the fourth binding domain of LRP1 (LRP1-IV) (Neels, J.G., *et al*. *J. Biol. Chem.* 2009, *274*, 31305–31311). In this work, LRP1-IV was expressed on the surface of *E. coli* using Autodisplay technology, to use whole cells for binding studies with ApoE, avoiding cumbersome protein purification. For this purpose an artificial gene for a fusion protein was constructed. The fusion protein consisted of an N-terminal signal peptide that directs the protein across the inner membrane of *E. coli*, the LRP1-IV domain, and the C-terminal autotransporter (AT). The AT facilitates the transport of the LRP1-IV to the surface of the cell (Jose, J., *et al*. *Microbiol. Mol. Biol. R.* 2007, *71*, 600–619). Surface display of LRP1-IV was verified by western blot of outer membrane preparations and flow cytometry of whole cells with a specific LPR1 antibody. Flow cytometric analysis also indicated that cells displaying LRP1-IV bind purified ApoE3 and ApoE3 labeled with PromoFluor-NHS 633. The assay as established enables to investigate the binding of ApoE3 variants to LPR1-IV or variants thereof and will allow determination of binding affinities.Our next step will be to test the binding of ApoE3 modified nanoparticles to surface displayed LPR1-IV. These nanoparticles could be used as carriers across the BBB for drugs which normally are not able to cross this barrier (Michaelis, K., *et al*. *J. Pharm. Exp. Ther.* 2006, *317*, 1246–1253; Zensi, A., *et al*. *J. Control Release* 2009, *137*, 78–86).

### 4.33. Maldi Detection of Alkaloids by Bithiophenic Matrices

A. Jaber ^1,2,^*, A. Schinkovitz ^1^, G. Kenfack ^1^, P. Richomme ^1^ and D. Seraphin ^1^

^1^ Laboratoire SONAS EA 921-IFR 149 QUASAV Substances d’Origine Naturelles et Analogues Structuraux, Université d’Angers 16 boulevard Daviers, Angers 49100, France

^2^ Laboratoire RDMNP-Recherche et Développement des Médicaments et des Produits Naturels, Université Libanaise, Faculté de Pharmacie, Hadath, Beyrouth, Liban

* Author to whom correspondence should be addressed; E-Mail: alijaber4891@hotmail.com.

Alkaloidsare bioactive secondary metabolites from plants exhibiting a wide range of pharmacological but also toxic effects.They are commonly used in many medicinal purposes applications. The present study investigates the improvement of the detection of alkaloidsby MALDI–MS in comparison to a recently developed and highly selective matrix molecule3-[5′-(methylthio)-2,2′-bithiophen-5-ylthio] propanenitrile (MT3P) (Schinkovitz, A., *et al*. *Anal. Bioanal. Chem.* 2012, *403*, 1697).

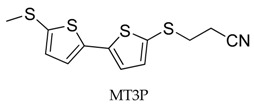


In this context several derivatives of MT3P have been synthesized and were tested on selected alkaloids. Obtained signal intensities were compared with those of MT3P. Furthermore the impact of using different solvents for matrix preparation was evaluated.The analyzed molecules showed comparable ionization properties.For MT3P increased sensitivity was observed when using ethanol as a matrix solvent.

### 4.34. HPLC-Assay with Ultraviolet Spectrometric Detection for Determination of Inhibitors of Plasma Amine Oxidase

Kira Mergemeier and Matthias Lehr *

Institute of Pharmaceutical and Medicinal Chemistry, University of Münster, Corrensstrasse 48, Münster 48149, Germany

* Author to whom correspondence should be addressed; E-Mail: lehrm@uni-muenster.de.

Plasma amine oxidase (PAO), also known as vascular adhesion protein-1 (VAP-1), is a quinone and copper dependent enzyme, which catalyzes the transformation of primary amines to aldehydes. Since PAO is required for transmigration of leukocytes through the endometrium, it is assumed to play an important role in inflammation-related diseases (Dunkel, P., *et al.* *Expert Opin. Ther. Patents* 2011, *21*, 1453–1471). Furthermore, PAO seems to be involved in ocular angiogenesis and tumor metastasis. Thus, inhibitors of this enzyme could be useful for the treatment of such conditions. For the evaluation of PAO inhibitors, usually assays are applied, which measure the transformation of benzylamine to benzaldehyde by UV-spectrometry (Artico, M., *et al.* *Eur J. Med. Chem.* 1992, *27*, 219–228; Shepard, E.M., *et al.* *Eur. J. Biochem.* 2002, *269*, 3645–3658).

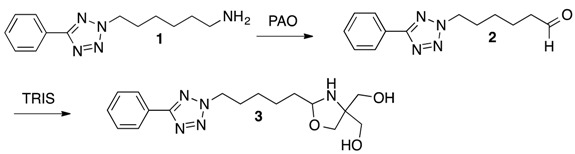


We have found that the phenyltetrazolylhexan-1-amine **1** is a better substrate for PAO than benzylamine. With this compound in hand, we established a novel HPLC-UV assay for the determination of inhibitors of PAO. Because the liberated aldehyde **2** gave a poor peak shape during HPLC chromatography due to hydrate formation, **2** was derivatized with TRIS (Tomori, E., *et al. Chromatographia* 1989, *27*, 228–232) to the oxazolidine **3**, which eluted as sharp peak. The method was validated and applied for the determination of the IC_50_-value of the known PAO inhibitor 2-(4-phenylphenyl)acetohydrazide (Artico, M., *et al.* *Eur J. Med. Chem.* 1992, *27*, 219–228; Shepard, E.M., *et al.* *Eur. J. Biochem.* 2002, *269*, 3645–3658).

### 4.35. Click Chemistry for Site Specific Fluorescence Labeling of Human Protein Kinase Ck2

Christian Nienberg ^1,^*, Kira Becher ^2^, Henning D. Mootz ^2^ and Joachim Jose ^1^

^1^ Institut für Pharmazeutischeund MedizinischeChemie, PharmaCampus, Westfälische Wilhelms-Universität Münster, Corrensstraße 48, Münster 48149, Germany

^2^ Institut für Biochemie, Westfälische Wilhelms-Universität, Wilhelm-Klemm-Straße 2, Münster 48149, Germany

* Author to whom correspondence should be addressed; E-Mail: christian.nienberg@uni-muenster.de.

Human CK2 is a heterotetrameric constitutively active serine/threonine protein kinase and plays an important role in today’s cancer research (Trembley, J.H., *et al*. *Biofactors* 2010, *36*, 187–195). The kinase is composed of two catalytic CK2α subunits and two regulatory CK2β subunits. Most protein-protein interaction (PPI) studies or screening assays are based on fluorescence detection and require the labelling of the target enzyme by a fluorophore. The catalytic subunit CK2α loses activity after labelling by commercial applications. Furthermore, the labelling ratio of the protein sample differs and is not exactly reproducible.

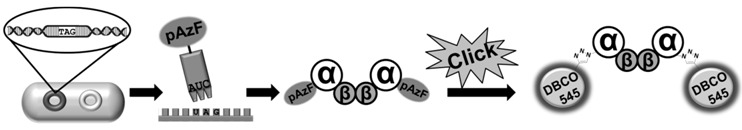


The solution for this problem was an incorporation of an unnatural amino acid into the CK2α subunit followed by a Strain-Promoted Alkyne-AzideCycloaddition (SPAAC) (Mbua, N.E., *et al*. *ChemBioChem* 2011, *12*, 1912–1921). Therefore, a suitable position in the sequence of CK2α was chosen and mutated to the ambernonsense DNA codon, TAG. By suppression of the mutation with an amber suppressor tRNA, the unnatural amino acid para-acidophenylalanine (pAzF) could be incorporated at this position (Chin, J.W., *et al*. *JACS* 2002, *124*, 9026–9027). Performing the SPAAC click reaction by the use of dibenzylcyclooctyne-fluor 545 (DBCO 545) led to a specifically labelled CK2α and CK2 holoenzyme. This specific kind of labelling does not impair the phosphorylation activity of the CK2α subunit alone nor the holoenzyme, which was evaluated by capillary electrophoresis. The innovatively labelled kinase in combination with the Autodisplay technology could be a significant advancement for inhibitor screening assays by flow cytometry and forCK2α/CK2β interaction studies (Jose, J. *et al*. *Microbiol. Mol. Biol. Rev.* 2007, *71*, 600–619).

### 4.36. Development of DPP-IV Inhibitors for the Treatment of Type 2 Diabetes

Salha Tawati *, Murray Robertson, Andrew Mccrone, Louise Young, Alan Harvey, Oliver Sutcliffe and Simon Mackay

Cell Biology Group and Medicinal Chemistry Research Group, Strathclyde Institute of Pharmacy and Biomedical Sciences, University of Strathclyde, 161 Cathedral Street, Glasgow G4 0RE, UK

* Author to whom correspondence should be addressed; E-Mail: salha.tawati@strath.ac.uk.

Type 2 diabetes mellitus is a worldwide chronic disorder which is characterised by insulin resistance and high blood glucose levels (Wu, J., *et al*. *J. Nanjing Med. Univ.* 2009, *23*, 228–235). The treatment of type 2 diabetes involves the use of traditional oral antidiabetic agents, such as sulphonylurea or glibenclamide, metformin, and/or insulin (Holt, R., *et al*. *Essential: Endocrinology and Diabetes (5th ed.)*; Blackwell Publishing: Oxford, UK, 2007). However, incidence of the disease is increasing in many parts of the world which means novel antidiabetic agents are needed.

Recently, new types of oral antidiabetic agents that inhibit DPP-IV have been developed and one has been approved for medical use. Collaboration between Strathclyde Innovations in Drug Research (SIDR) and the Drug Discovery Portal (DDP) using a combination of virtual and high throughput screening identified a novel hit (AM11) against DPP-IV. The aim of this project was to modify AM11 to see if inhibition against DPP-IV could be improved.

Compounds with a range of activities against DPP-IV were prepared, but all had comparable or lower activity than AM11. Docking studies were performed to explain the structure-activity relationship profiles of the different libraries prepared.

### 4.37. Preparation of Semisynthetic Macrocycles Using Split Inteins

Shubhendu Palei ^1,^* and Henning D. Mootz ^1,2^

^1^ Institute of Biochemistry, University of Muenster, Wilhelm Klemm Strasse 2, Muenster 48149, Germany

^2^ International NRW Graduate School of Chemistry (GSC-MS), Wilhelm Klemm Strasse 2, Muenster 48149, Germany

* Author to whom correspondence should be addressed; E-Mail: s_pale01@uni-muenster.de.

Cyclic peptides are best representatives of peptide based drugs because of their unique properties like stability towards pH change, heat denaturation, protease degradation as compared to their linear counterparts. The cyclic backbone also has less conformational flexibility and small entropy which increase their binding affinity towards the peptide or protein interface (Driggers, E.M., *et al*. *Nat. Rev. Drug Discov.* 2008, *7*, 608–624). The demand of using semisynthetic macrocycles for therapeutic applications is getting more attention as it can give access to diverse functionalities and unnatural amino acids through the synthetic part. Here in, we have developed a novel method to make semisynthetic macrocycles which is accessible to any unnatural amino acid or functionality. We make use of protein trans-splicing followed by bioorthogonal oxime ligation to join two ends of synthetic and recombinant parts of the macrocycle. The synthetic part is accessible through solid phase peptide synthesis whereas the recombinant part is expressed in *E. coli.* Therefore, the semisynthetic macrocyclic backbone can simultaneously be randomized recombinantly along with any therapeutically important non-natural synthetic moiety. We have shown the synthesis of not only a number of macrocycles of different ring sizes, but also of a number of semisynthetic lipopeptides that represent structural analogues of the nonribosomal lipopeptide antibiotic daptomycin (Robbel, L., *et al*. *J. Bio. Chem.* 2010, *285*, 27501–27508). This method promises a powerful tool for generation and selections of wide varieties of semisynthetic macrocycles of therapeutic importance.

### 4.38. Hemisynthesis of Natural Formylated Tocotrienols

K. Alsabil, J.-J. Helesbeux and D. Seraphin *

SONAS, University of Angers, 42 rue Georges Morel, Beaucouzé 49070, France

* Author to whom correspondence should be addressed; E-Mail: denis.seraphin@univ-angers.fr.

Garcinia is the most numerous genus of the Clusiaceae family with about 400 species widely distributed in tropical Asia, Africa, New Caledonia and Polynesia (Waterman, P.G. *Phytochemistry* 1986, *25*, 17). Garcinia species are known to be rich in oxygenated derivatives (Peres, V., *et al*. *Phytochemistry* 2000, *55*, 683), some of them exhibiting various biological activities such as anti-inflammatory (Gopalakrishnan, C., *et al*. *Ind. J. Exp. Biol.* 1980, *18*, 843) and antioxidant properties (Hay, A.E., *et al*. *J. Nat. Prod.* 2004, *67*, 707).

The cyclohexane-soluble extract of the stem bark of *Garcinia virgata* was found to exhibit significant antioxidant effects, based on the scavenging of the stable DPPH free radical. Amongst the different secondary metabolites isolated from this extract, we obtained two new vitamin E analogs, 5-formyl- and 7-formyl-δ-tocotrienols **1** and **2** (Merza, J., *et al*. *Phytochemistry* 2004, *65*, 2915). Unfortunately, for both of them, available material was insufficient to evaluate their antioxidant potential. At that time, we were also unable to record all the spectral data of compound **2**.

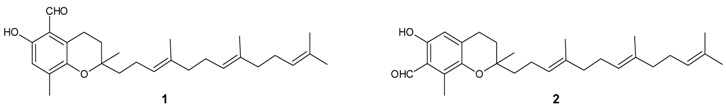


In the present work we decided to achieve the semisynthesis of these two analogs of vitamin E using Duff formylation starting from δ-tocotrienol, we previously isolated from *Mammea neurophylla*. As this reaction is regiospecific, synthetic pathway to compound **2** required a protection of C-5 position of the chromanol.

### 4.39. Acylphloroglucinol Derivatives: Synthesis, Anti -Ages, Anti-Oxydant and Vascular Activities

Isabelle Baglin ^1,^*, Sangeetha Thirumaran ^1^, Lizeth Bodero ^1^, Séverine Derbré ^1^, Patricia Blanchard ^1^, Dimitri Bréard ^1^, Pascal Richomme ^1^, Emilie Vessières ^2^, Daniel Henrion ^2^ and Denis Séraphin ^1^

^1^ IFR 149 QUASAV, SONAS, UFR Pharmacie, 16, Bd Daviers, Angers 49100, France

^2^ Laboratoire de Biologie Neurovasculaire et Mitochondriale Intégrée, Inserm U771, CNRS UMR 6214, Université d'Angers, Angers, France

* Author to whom correspondence should be addressed; E-Mail: isabelle.baglin@univ-angers.fr.

Our laboratory is interested in natural products therapeutic promotion, in that way we particularly studied antiAGEs activities of natural, semisynthetic and synthetic compounds (Derbré S., *et al*. *Anal. Bioanal. Chem.* 2010, *398*, 1747–1758). Advanced Glycation Endproducts (AGEs) retained scientifics attention because the glycated proteins generated by Maillard reaction are implicated in many diseases such as diabetes and neurological diseases (Alzheimer, Parkinson…) (Li, J., *et al*. *J. Neurol. Sci.* 2012, *317*, 1–5). AGEs accumulation is also associated with cardiovascular dysfunction including atherosclerotic plaque formation, decreased vascular elasticity, endothelial dysfunction and hypertension (Li, J., *et al*. *J. Neurol. Sci.* 2012, *317*, 1–5).

Acylphloroglucinols derived from secondary metabolites in Clusiaceae family. These compounds attracted our attention because of their antiAGEs activities, among them benzophenones are particularly interesting. The phloroglucinol skeleton, poorly toxic and well known, was selected to design three new series of aminophloroglucinols and acylaminophloroglucinols. This choice was guided by the intrinsic antiAGEs activity of phloroglucinol and its particular reactivity towards secondary amines.

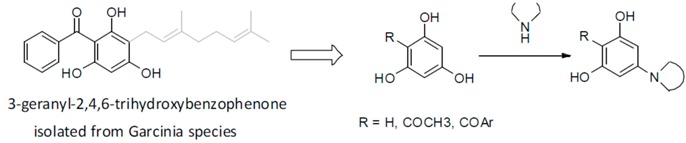


As ALT711, the reference in AGE breakers demonstrated effect on vasoactivity (Freidja, M.L., *et al*. *Diabetes* **2012**, *61*, 1562–1572), we managed to determine the influence of our derivatives on vascular activity. Our previous study highlighted the antiAGEs and/or antioxidant properties of some derivatives. So by analysing all these results we expected to confirm that antiAGEs and/or antioxidant activities would correlate with vascular properties.

### 4.40. Casein Kinase 1 Inhibitors: Synthesis and Biological Evaluation of 2,3-Diarylimidazo[1,2-a]-Pyrazines as Antileishmanial Agents

Marc-Antoine Bazin ^1^, Fabrice Pagniez ^2^, Guillaume Rivière ^1^, Lizeth Bodero ^1^, Sophie Marhadour ^1^, Carine Picot ^1,2^, Sandrine Ruchaud ^3^, Stéphane Bach ^3^, Patrice Le Pape ^2^ and Pascal Marchand ^1,^*

^1^ Laboratoire de Chimie Thérapeutique, Université de Nantes, Nantes Atlantique Universités, Cibles et Médicaments des Infections et du Cancer, IICiMed UPRES EA 1155, UFR des Sciences Pharmaceutiques et Biologiques, 1 rue Gaston Veil, Nantes 44035, France

^2^ Laboratoire de Parasitologie et Mycologie Médicale, Université de Nantes, Nantes Atlantique Universités, Cibles et Médicaments des Infections et du Cancer, IICiMed UPRES EA 1155, UFR des Sciences Pharmaceutiques et Biologiques, 1 rue Gaston Veil, Nantes 44035, France

^3^ CNRS, Protein Phosphorylation & Human Disease group, Station Biologique, Place Georges Teissier, Roscoff 29680, France

* Author to whom correspondence should be addressed; E-Mail: pascal.marchand@univ-nantes.fr.

According to a recent report from the World Health Organization (WHO), leishmaniases—visceral leishmaniasis (VL), cutaneous leishmaniasis (CL), and mucocutaneous leishmaniasis (MCL)—collectively affect 12 million people in 98 countries, 350 million more are at risk of infection and 40000 deaths are attributed to leishmaniases each year (Hussain, H., *et al*. *Chem. Rev.* 2014, *114*, 10369–10428). Currently, there are no effective vaccines and a number of drugs are used in the treatment of these parasitic infections: pentavalent antimonials, amphotericin B, miltefosine, pentamidine, paromomycin, and sitamaquine (Hussain, H., *et al*. *Chem. Rev.* 2014, *114*, 10369–10428). Unfortunately, most of them cause side effects and high toxicities, and an inevitable resistance has developed in recent times in *Leishmania* parasites. Consequently, there is an urgent need to speed up the development of a new generation of more effective and safe antileishmanials.

In the course of our ongoing synthetic and screening programs for new biologically active imidazo[1,2-*a*]azines, we decided to develop bioisostere analogues of the previously described 2,3-diarylimidazo[1,2-*a*]pyridines **I** as antileishmanial agents.

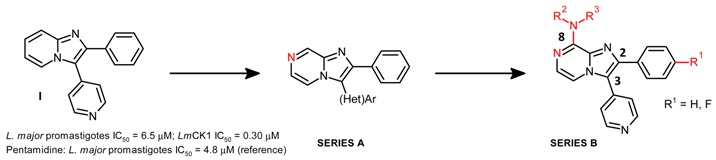


Imidazo[1,2-*a*]pyrazines have been gaining attention in drug discovery but no biological application related to *Leishmania* disease was found in the literature (Rachidi, P., *et al*. *Eur. J. Med. Chem.* 2012, *58*, 543–556). We planned to explore such scaffold for the design of antileishmanial agents associated with *L. major* CK1 kinase inhibition, validated as potential molecular target for antiparasitic drug development (Goel, R., *et al*. *Org. Biomol. Chem.* 2015, *13*, 3525–3555).

### 4.41. Synthetic 3-Deoxyanthocyanidin Analogues of Delphinidin as Potential Vasorelaxant Agents

Samuel Legeay ^1^, Sébastien Faure ^1^, Jean-Jaques Helesbeux ^2,^*, Yannick Abatuci ^2^, Olivier Duval ^2^, Ramaroson Andriantsitohaina ^1^

^1^ SOPAM INSERM UMR S1063, University of Angers, 4 Rue Larrey, 49933 Angers Cedex 9 France

^2^ SONAS, University of Angers, 42 rue Georges Morel, Beaucouzé 49070, France

* Author to whom correspondence should be addressed; E-Mail: jean-jacques.helesbeux@univ-angers.fr.

A greater reduction in cardiovascular risk and vascular protection associated with diet rich in polyphenols are generally accepted; however, the molecular targets for polyphenols effects remain unknown. Recently, a study using ERα deficient mice showed that endothelium-dependent vasorelaxation induced by delphinidin, an anthocyanin found in numerous fruits or vegetables, is mediated by ERα (Chalopin, M., *et al*. *PLoS ONE* 2010, *5*, e8554). As a matter of fact, this natural secondary metabolite is able to induce endothelial vasodilatation in aorta from ERα Wild-Type but not from Knock-Out mice, by activation of nitric oxide (NO) pathway in endothelial cells. Besides, all the data obtained during this study provided evidence that delphinidin, through direct interaction with ERα, activated molecular pathways including Src, ERK1/2, eNOS, leading to endothelial NO production, accounting for vasorelaxation.

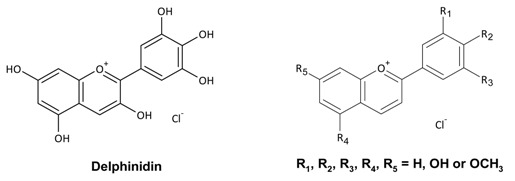


Anthocyanidins, and delphinidin particularly, have a low bioavailability (Manach, C., *et al*. *Am. J. Clin. Nutr.* 2005, *81*, 230S–242S), and suffer from a pH-dependent instability. In the present study, we aimed at better understanding the mode of action of delphinidin and also developing new analogues with optimized biological and physicochemical properties. To do so, we synthesized 3-deoxyanthocyanidin derivatives, supposedly more stable than the parent series (Fleschhut, J., *et al*. *Eur. J. Nutr.* 2006, *45*, 7; Chassaing, S. *Eur. J. Org. Chem.* 2007, *15*, 2438) and modified the substitution pattern of the remaining oxygenated positions of the flavylium backbone. The vasorelaxant potential of each analogue was then evaluated by myography on mice aorta in comparison with delphinidin activity.

## 5. Conclusions

To honor the work of PhD students and young scientists, two awards were attributed for the best poster presentation, and for the best oral flash presentation. The first award was attributed to Jana K. Böcker (Institute of Biochemistry, University of Münster) for her poster entitled “Generation of a genetically encoded, photoactivable intein for the controlled production of cyclic peptides”. For the best flash presentation, Anke Jakobs (Institute of Biochemistry, University of Münster) received the second award. Her oral presentation was entitled “Inhibition of C/EBPB by the sesquiterpene lactone helenalinacetate”. The 24th Conference of GP2A will take place next summer, during the last week of August 2016, in Angers (Region Pays-de-la-Loire, France). The ChemBioInteract network will continue to explore new therapeutic targets and then to build new International Collaborative Research Projects (ICRP).

